# TRIM21 and PHLDA3 negatively regulate the crosstalk between the PI3K/AKT pathway and PPP metabolism

**DOI:** 10.1038/s41467-020-15819-3

**Published:** 2020-04-20

**Authors:** Jie Cheng, Yan Huang, Xiaohui Zhang, Yue Yu, Shumin Wu, Jing Jiao, Linh Tran, Wanru Zhang, Ran Liu, Liuzhen Zhang, Mei Wang, Mengyao Wang, Wenyu Yan, Yilin Wu, Fangtao Chi, Peng Jiang, Xinxiang Zhang, Hong Wu

**Affiliations:** 1 The MOE Key Laboratory of Cell Proliferation and Differentiation, School of Life Sciences, Peking-Tsinghua Center for Life Sciences, Beijing Advanced Innovation Center for Genomics, Peking University, Beijing, 100871 China; 2 Beijing National Laboratory for Molecular Sciences, Key Laboratory of Bioorganic Chemistry and Molecular Engineering of Ministry of Education, College of Chemistry and Molecular Engineering, Peking University, Beijing, 100871 China; 3 Department of Molecular and Medical Pharmacology, University of California, Los Angeles, Los Angeles, CA 90095 USA; 4 Department of Molecular, Cell and Developmental Biology, University of California, Los Angeles, Los Angeles, CA 90095 USA; 5 School of Life Sciences, Tsinghua University, Beijing, 100084 China

**Keywords:** Biochemistry, Cancer metabolism

## Abstract

PI3K/AKT signaling is known to regulate cancer metabolism, but whether metabolic feedback regulates the PI3K/AKT pathway is unclear. Here, we demonstrate the important reciprocal crosstalk between the PI3K/AKT signal and pentose phosphate pathway (PPP) branching metabolic pathways. PI3K/AKT activation stabilizes G6PD, the rate-limiting enzyme of the PPP, by inhibiting the newly identified E3 ligase TIRM21 and promotes the PPP. PPP metabolites, in turn, reinforce AKT activation and further promote cancer metabolic reprogramming by blocking the expression of the AKT inhibitor PHLDA3. Knockout of TRIM21 or PHLDA3 promotes crosstalk and cell proliferation. Importantly, *PTEN* null human cancer cells and in vivo murine models are sensitive to anti-PPP treatments, suggesting the importance of the PPP in maintaining AKT activation even in the presence of a constitutively activated PI3K pathway. Our study suggests that blockade of this reciprocal crosstalk mechanism may have a therapeutic benefit for cancers with PTEN loss or PI3K/AKT activation.

## Abstract (web summary)

The PI3K/AKT signalling pathway regulates cancer metabolism. Here, the authors show a reciprocal positive feedback crosstalk between the PI3K/AKT and pentose phosphate pathway which promotes tumourigenesis.

## Introduction

Metabolic reprogramming is one of the hallmarks of human cancers^1^. Unlike normal cells, cancer cells catabolize high levels of glucose for lactate production, even when oxygen is abundant, through a phenomenon called aerobic glycolysis or the “Warburg effect“^2^. Cancer cells express pyruvate kinase 2 (PKM2), a growth signal-sensitive form of the rate-limiting enzyme in the last step of glycolysis^3^, leading to the accumulation of glycolytic intermediates. The accumulated glycolytic intermediates can branch into the PPP for nucleotide biosynthesis and NADPH production, which is important for cell proliferation and redox state maintenance^4^. The PPP is highly upregulated in cancers to meet the needs of uncontrolled cell proliferation^5,6^. Cancer cells show increased sensitivity to PPP inhibition^7^ and chemotherapies that inhibit nucleotide biosynthesis^8^. However, the mechanisms that orchestrate glycolysis and the PPP in normal and cancer cells are largely unknown.

The PI3K/AKT pathway is one of the most commonly altered signaling pathways in human cancers^9–11^. PI3K/AKT promotes the Warburg effect by increasing the expression and membrane translocation of glucose transporters and the activities of enzymes involved in glycolysis. PTEN is the major tumor suppressor that antagonizes the PI3K/AKT pathway via its lipid phosphatase activity^12–14^ and therefore exerts an “anti-Warburg” effect. Indeed, overexpression of the murine *Pten* gene in a transgenic model decreased glycolysis and increased respiration^15^. However, since PTEN possesses both lipid and protein phosphatase activities as well as phosphatase-independent activities^14^, it is not clear whether the metabolic phenotype observed in the *Pten* overexpression model is solely due to its lipid phosphatase or anti-PI3K/AKT activity. It is also not clear whether PTEN loss or PI3K/AKT activation controls the PPP branching pathway in cancer metabolic reprogramming.

To answer these questions, we genetically knock-in two cancer-associated PTEN point mutations into the endogenous *Pten* gene in embryonic stem cells (mES): the C124S mutation, which results in a phosphatase-dead phenotype, and the G129E mutation, which results in a lipid phosphatase-dead and protein phosphatase-active phenotype. These two mutant lines, together with the parental WT and *Pten* null lines^16^, allow us to genetically separate the lipid and protein phosphatase activities as well as the phosphatase-independent activity of PTEN without perturbing its level (Supplementary Fig. 1A). Using this true isogenic system, we conduct metabolic chase analyses on these four cell lines and in an ES cell system that mimics cancer metabolism^17,18^.

To confirm the relevance of our findings in vivo and in human cancers, we also use the *Pten* null prostate cancer and T-ALL mouse models, as they closely mimic the clinical features of these human cancers with high frequencies of PTEN mutation and PI3K pathway activation^19–22^, as well as the PTEN null human prostate cancer and T-ALL cell lines.

Here, we report a reciprocal crosstalk mechanism between the PI3K/AKT pathway and the PPP in *Pten* mutant mES cells, which is further confirmed in in vivo cancer models and human cancer cells with PTEN loss. PTEN loss or PI3K/AKT activation promotes a shift of glycolytic intermediates to the PPP branching pathway by stabilizing the rate-limiting enzyme G6PD. PPP metabolites, in turn, provide positive feedback and reinforce PI3K/AKT activation via negative regulation of the AKT inhibitor PHLDA3. These positive feedback mechanisms between metabolic pathways and cell signaling may have important therapeutic implications for cancers with PTEN loss and PI3K/AKT activation.

## Results

### PI3K activation decouples glycolysis and TCA cycle

**Fig. 1:**
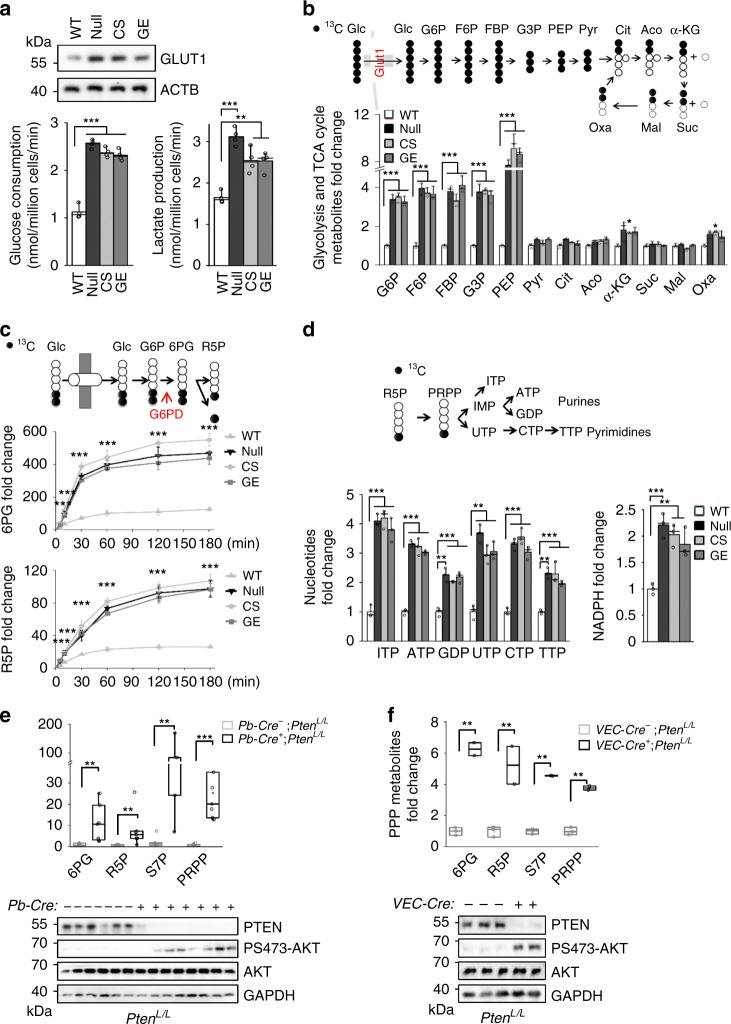
PTEN loss or PI3K activation promotes glycolysis and PPP. **a** Loss of the PTEN lipid phosphatase activity increases the GLUT1 levels (upper panel), glucose consumption and lactate production in the *Pten* null, CS, and GE mES cells compared with the isogenic WT cells. **b** Upper panel, a schematic illustrating [U-^13^C] glucose metabolism; lower panel, loss of the PTEN lipid phosphatase activity increases the levels of ^13^C-labeled glycolytic intermediates from G6P to PEP in the *Pten* null, CS, and GE mES cells compared with the isogenic WT cells. Glucose-6-phosphate (G6P), fructose-6-phosphate (F6P), fructose-1,6-bisphosphate (FBP), gyyceraldehyde-3-phosphate (G3P), phosphoenolpyruvate (PEP), pyruvate (Pyr), citrate (Cit), aconitate (Aco), α-ketoglutarate (α-KG), succinate (Suc), malate (Mal), oxaloacetate (Oxa). **c** Upper panel, a schematic illustrating [1,2-^13^C] glucose tracing into the oxidative arm of the PPP; lower panel, faster and higher levels of labeled 6-phosphogluconate (6PG) and ribose-5-phosphate (R5P) in the *Pten* null, CS, and GE mES cells compared with the WT cells. **d** Upper panel, a schematic illustrating [1,2-^13^C] glucose tracing into the nucleotide biosynthesis pathway; lower panel, increased levels of labeled nucleotides and NADPH production in the *Pten* null, CS, and GE mES cells compared with the WT cells. **e**,**f** Upper panels, increased PPP metabolites in the *Pten* null prostate cancer and T-ALL mouse models. Metabolites in the PPP were extracted from the anterior lobes of the *Pb-Cre*^*−*^*;Pten*^*L/L*^ and *Pb-Cre*^*+*^*;Pten*^*L/L*^ mice (**e**) or the thymus of the *VEC*^−^*Cre*^*−*^*;Pten*^*L/L*^ and *VEC*^−^*Cre*^*+*^*;Pten*^*L/L*^ mice (**f**) by box plots. Median and quartile values are provided by the central line and box boundaries. Whiskers show min to max values. Sedoheptulose-7-phosphate (S7P), phosphoribosyl pyrophosphate (PRPP). *n* = 7 (**e**), *n* = 3 (**f**), numbers of independent mouse samples. **a**–**d**, cell extracts from *Pten* mutant cell lines were prepared and analyzed using LC-MS. Data are presented as fold changes and the mean ± SD and were compared with the WT cells. Each experiment was performed *n* = 4 (**a**) and *n* = 3 (**b**–**d**) independent times. *p < 0.05, **p < 0.001, ***p < 0.001, based on Student’s *t* test (two-sided ANOVA). See also Supplementary Fig. 1. Source data are provided as a Source Data file.

To fully explore the roles of PTEN in regulating cell metabolism, we measured glucose consumption in isogenic WT, null, CS and GE mES cells under standard ES culture conditions and found that all three *Pten* mutant lines expressed higher levels of GLUT1 and consumed more glucose than the WT line (Fig. 1a, upper and lower left panels). The *Pten* mutant lines also secreted more lactate and had higher ECAR rates than the WT line (Fig. 1a, lower right panel; Supplementary Fig. 1B). Since all three *Pten* mutant lines lacked lipid phosphatase activity and the PI3K inhibitor PKI-587 can revert the aforementioned phenotypes (Supplementary Fig. 1A, C), this result suggests that PTEN regulates the Warburg effect by antagonizing PI3K activity.

We then investigated the fate of uniformly labeled [U-^13^C]-glucose by measuring the metabolic intermediates of glycolysis and the TCA cycle (Fig. 1b, upper panel)^23^. Although no obvious differences were observed in the TCA metabolites between the WT and *Pten* mutant lines, the levels of glycolytic intermediates from G6P to PEP were significantly increased in all *Pten* mutant lines (Fig. 1b, lower panel), and this change was reversed by PKI-587 treatment (Supplementary Fig. 1D). In contrast, pyruvate (pyr), the downstream metabolite of PEP whose production is catalyzed by the rate-limiting enzyme PKM2 before the TCA cycle^8,24^, showed no significant changes (Fig. 1b, lower panel; Supplementary Fig. 1D). PKM2 Y105 phosphorylation is known to inhibit PKM2 activity and promote the Warburg effect and tumor growth^3,25^. We observed significantly increased PKM2 Y105 phosphorylation and decreased PKM activity in all *Pten* mutant lines (Supplementary Fig. 1E); these changes were reverted by re-expressing WT PTEN or PKI-587 treatment but not by re-expressing the phosphatase-dead CS PTEN, suggesting that the loss of PTEN lipid phosphatase activity or the activation of the PI3K/AKT pathway may control the Warburg effect, at least in part, by inhibiting PKM2 activity and decoupling glycolysis and the TCA cycle.

### PI3K activation diverts glycolytic intermediates to PPP

Since PI3K activation led to 4-8-fold increases in the levels of glycolytic intermediates from G6P to PEP (Fig. 1b), we next investigated whether these accumulated metabolites would flow to the branching metabolic pathways. We traced [1, 2-^13^C]-glucose for the indicated times and quantified the amount of labeled glucose entering the oxidative arm of the PPP^23^. As shown in Fig. 1c, [1, 2-^13^C]-glucose incorporated into 6PG and R5P much faster and at higher levels in the *Pten* mutant lines than in the WT line. Furthermore, intermediates from both the oxidative and nonoxidative arms, traced by [U-^13^C]-glucose, PPP-associated nucleotides and NADPH production were significantly increased in the *Pten* mutant lines compared with the WT line (Fig. 1d; Supplementary Fig. 1F).

Consistent with the results from the isogenic ES cell lines, the PPP metabolites were significantly higher in the *Pten* null in vivo cancer models compared with their *Cre*^*−*^ WT littermates (Fig. 1e,f). Therefore, PTEN loss or PI3K activation drives the Warburg effect, at least in part, by reducing PKM2 activity and diverting accumulated glycolytic intermediates to the PPP to support the rapid proliferation of the *Pten* mutant cells.

### PI3K/AKT activation promotes PPP by stabilizing G6PD

**Fig. 2:**
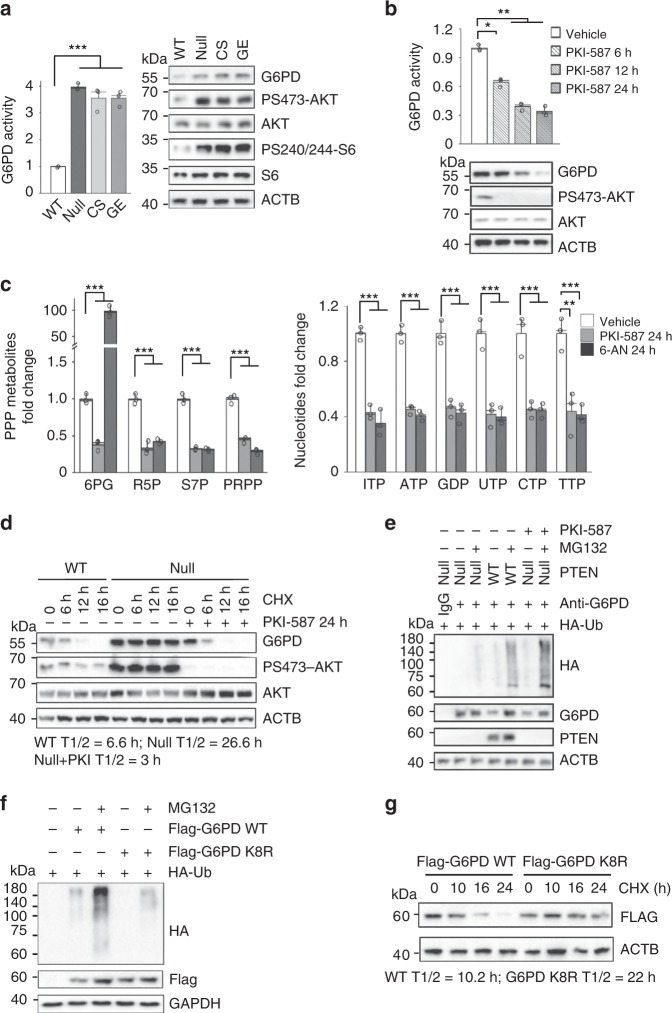
PI3K/AKT activation promotes PPP by stabilizing G6PD. **a, b** The enzymatic activities and protein levels of G6PD were increased in the *Pten* null, CS and GE mES cells compared with the WT cells, and decreased after PKI-587 (1 µM) treatment in the *Pten* null mES cells. **c** PPP metabolites and nucleotide production were decreased by PKI-587 or 6-AN treatment. The *Pten* null mES cells were switched to medium containing [U-^13^C] glucose and continuously cultured for 12 h, with or without PKI-587 (1 µM) or 6-AN (100 nM) for the indicated time periods. **d** The half-lives of G6PD in the *Pten* WT and null mES cells were measured after PKI-587 (1 µM) or vehicle treatment for 24 h, or treatment with CHX (100 µg/ml) for the indicated time periods. **e** PTEN controls ubiquitination-mediated degradation of the endogenous G6PD. *PTEN* WT and null HEK293T cells were transfected with the *HA-Ubiquitin* expression plasmids. Twenty-four hours later, cells were incubated with or without MG132 (5 μM) or PKI-587 (1 µM) for 24 h. Total cell lysates were immunoprecipitated with an anti-G6PD antibody. **f** HEK293T cells were cotransfected with the *HA-ubiquitin*, *Flag-G6PD WT* or *Flag-G6PD K8R* expression plasmids. Twenty-four hours later, the cells were incubated with or without MG132 (5 µM) for 16 h. Total cell lysates were harvested, immunoprecipitated with a Flag antibody. **g** The half-lives of exogenous WT and K8R G6PD were measured after CHX (100 µg/ml) treatment for the indicated time periods. Data are presented as the mean ± SD and compared with the WT or untreated cells. *n* = 3, each experiment was performed four independent times. *p < 0.05, **p < 0.001, and ***p < 0.001, based on Student’s *t* test (two-sided ANOVA). See also Supplementary Fig. 2 and Supplementary Table 1. Source data are provided as a Source Data file.

Although the underlying mechanisms of PI3K-controlled glucose transport and glycolysis have been well studied, little is known about how PI3K controls the PPP branching metabolic pathway^9,26,27^. G6PD is the rate-limiting enzyme in the PPP. While there was no significant difference in the *G6pd* mRNA levels (Supplementary Fig. 2A), we did detect ~2-4-fold increases in the G6PD protein levels and enzymatic activities in all *Pten* mutant mES lines (Fig. 2a), which could be reverted by the PI3K inhibitor PKI-587 but not the MEK inhibitor PD0325901 (Fig. 2b, c; Supplementary Fig. 2B). The potency of PKI-587 was comparable to that of 6-AN, an inhibitor of G6PD, except 6-AN can increase the 6PG levels due to its inhibitory effects on both G6PD and PGD^28^ (Fig. 2c). These results suggest that PI3K pathway activity is responsible for regulating the G6PD protein level and activity.

Since PI3K/AKT regulates the G6PD protein level instead of its mRNA level, we measured the half-life of the G6PD protein in the WT and *Pten* null mES cells. PTEN loss significantly prolonged the half-life of the G6PD protein from 6.6 h to 26.6 h, while PKI-587 treatment completely inhibited this effect (Fig. 2d). To test whether PI3K/AKT affects the stability of G6PD through the ubiquitin-mediated degradation pathway, we expressed *HA-ubiquitin* in *PTEN* WT and null 293T cells or *Myc-PTEN*, *Flag-G6PD* and *HA-ubiquitin* in 293T cells and treated the cells without or with MG132. PTEN expression or inhibition of PI3K activity significantly increased both endogenous and exogenous levels of ubiquitinated G6PD, and decreased the levels of G6PD protein, which could be reverted by MG132 treatment (Fig. 2e, Supplementary Fig. 2C).

To identify the specific residues responsible for G6PD ubiquitylation, we conducted mass spectrometry analysis and found 8 potential ubiquitylated lysines (Supplementary Fig. 2D). Mutating all 8 lysine (K) residues to arginine (R) significantly decreased the ubiquitination of K8R G6PD and prolonged its half-life compared with that of WT G6PD (Fig. 2f, g). Together, these results suggest that PI3K activation promotes the PPP branching pathway by inhibiting ubiquitin-mediated degradation of G6PD.

### TRIM21 is the E3 ligase responsible for G6PD degradation

**Fig. 3:**
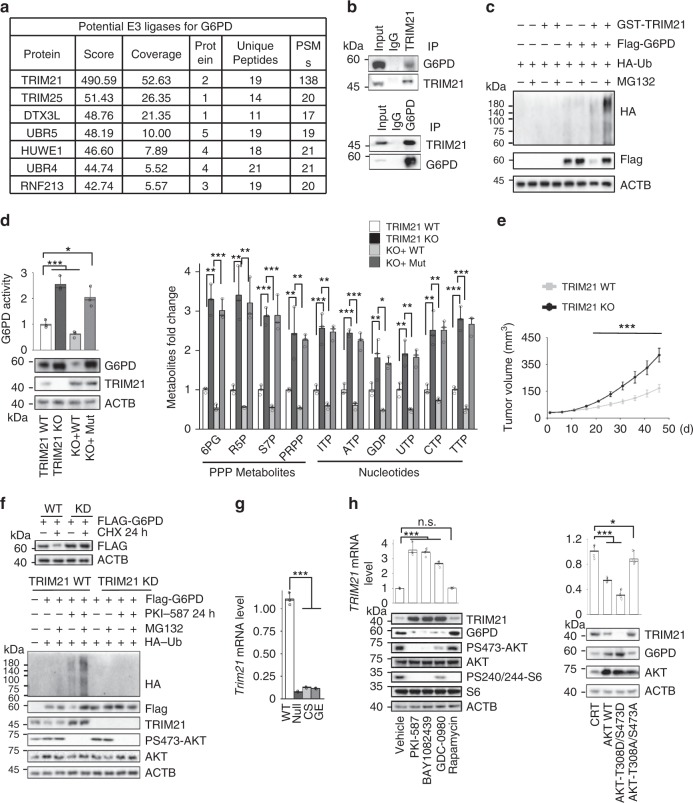
TRIM21 is responsible for PI3K/AKT-regulated G6PD stability. **a** The potential G6PD E3 ligases identified by affinity MS. **b** TRIM21 interacts with endogenous G6PD. PC3 cell lysates were reciprocally immunoprecipitated and immunoblotted with indicated antibodies. **c** TRIM21 controls G6PD ubiquitination. HEK293T cells were cotransfected with indicated plasmids; 24 h later, incubated with or without MG132 (5 µM) for 16 h, Total cell lysates were immunoprecipitated with a Flag antibody and immunoblotted with the indicated antibodies. **d** The enzymatic activity and protein levels of G6PD (left panel) and PPP metabolites and nucleotides (right panel) in the *TRIM21* WT and knockout A549 cells, and knockout cells re-expressing the WT or E3 ligase-dead mutant *TRIM21* plasmid. *n* = 6 independent xenografts. **e** The growth kinetics of the isogenic *TRIM21* WT and knockout A549 cell-derived xenografts in vivo. Equal numbers of *TRIM21* WT and knockout cells were implanted into the bilateral flanks of nude mice. Relative tumor volumes are presented with growth kinetics. **f** TRIM21 controls G6PD ubiquitination-mediated degradation. HEK293T cells were transfected with si-scramble (WT) or siTRIM21 (KD) RNAs and 2 days later, cells were treated with or without CHX for 24 h (upper) or transfected with *HA-ubiquitin* and *Flag-G6PD* expression plasmids (lower). After 24 h, the cells were treated with or without PKI-587 for 24 h or MG132 for 16 h and total cell lysates were immunoprecipitated with a Flag antibody and immunoblotted with the indicated antibodies. **g** The *Trim21* mRNA levels are lower in the null, CS and GE mES cells than in WT cells. **h** Left, PI3K/AKT inhibitors control *TRIM21* and G6PD levels. PC3 cells were treated with PKI-587 (1 µM), BAY1082439 (5 µM), GDC-0980 (40 µM), GDC-0068 (10 µM) or rapamycin (100 nM) for 24 h. Right panel, HEK293T cells were transfected with AKT-WT, AKT-T308D/S473D or AKT-T308A/S473A plasmids. Metabolites were measured using LC-MS. Data are presented as fold changes and the mean ± SD and were compared with WT or untreated cells. Each experiment was performed *n* = 3 (**d**, **g**) and *n* = 4 (**h**) independent times. *p < 0.05, **p < 0.001, and ***p < 0.001, based on Student’s *t* test (two-sided ANOVA). See also Supplementary Fig. 3. Source data are provided as a Source Data file.

To identify the E3 ligase responsible for G6PD degradation, we expressed *Flag-G6PD* in the *PTEN* null prostate cancer PC3 cells and performed a mass spectrometric analysis of G6PD-associated proteins. Among the seven potential E3 ligases identified, TRIM21 received the highest score (Fig. 3a). Reciprocal coimmunoprecipitation of endogenous proteins confirmed the physical interaction between G6PD and TRIM21 in vivo (Fig. 3b), and this interaction was not influenced by PKI-587 treatment (Supplementary Fig. 3A). To determine whether TRIM21 participates in G6PD ubiquitylation, we expressed *GST-TRIM21*, *Flag-G6PD* and *HA-ubiquitin* in 293T cells followed by treatment without or with MG132. As shown in Fig. 3c, *TRIM21* overexpression increased the G6PD ubiquitination, accompanied by decreased levels of the G6PD protein that could be reverted by MG132.

To further confirm the functional role of TRIM21 in regulating the G6PD protein levels, we either overexpressed or knocked down *TRIM21* in PC3 cells. As predicted for an E3 ligase, the TRIM21 protein levels were inversely correlated with the levels of G6PD (Supplementary Fig. 3B). We also knocked out *TRIM21* and found that *TRIM21* knockout could increase both the protein level and enzymatic activity of G6PD, as well as the levels of PPP metabolites (Fig. 3d). Re-expression of WT *TRIM21*, but not the E3 ligase-dead mutant (C16A, C31A and H33W)^29^, in *TRIM21* knockout cells could significantly reduce the levels of G6PD as well as the PPP metabolites (Fig. 3d), strongly supporting the notion that TRIM21 is the E3 ligase that controls G6PD degradation. Increased PPP activity in the *TRIM21* knockout cells also led to increased cell growth and tumor volume in vitro and in vivo, respectively, demonstrating the important role of TRIM21 in negatively regulating the PPP and cell growth (Fig. 3e; Supplementary Fig. 3C, D).

The G6PD protein is very stable in *TRIM21* knockdown and knockout cells, and its half-life and ubiquitination were not altered by PKI-587 or MG132 treatment (Fig. 3f and Supplementary Fig. 3E, F), suggesting that TRIM21 may be the target of PI3K/AKT signaling. Consistent with this notion, we found that the *Pten* mutant mES cells had much lower *Trim21* mRNA levels than the WT cells (Fig. 3g). After treatment of the PTEN null PC3 cells with anti-PI3K/AKT inhibitors as well as pan-mTOR inhibitors, but not the mTOR1 inhibitor rapamycin, could promote *Trim21* expression and increase the TRIM21 protein levels while reducing the G6PD levels (Fig. 3h, left panel; Supplementary Fig. 3G), suggesting an AKT/mTORC2-dependent mechanism. Overexpression of WT AKT, especially its constitutively activated form (AKT-T308D/S473D) but not its inactivated form (AKT-T308A/S473A), inhibited *TRIM21* transcription and decreased its protein levels, accompanied by increased G6PD protein levels (Fig. 3h, right panel). These results suggest that AKT/mTORC2 activities are responsible for suppressing *Trim21* expression and increasing the G6PD protein levels and activity in the *Pten* mutant ES cells.

### PI3K/AKT regulates TRIM21 and PPP in mouse and human cancers

**Fig. 4:**
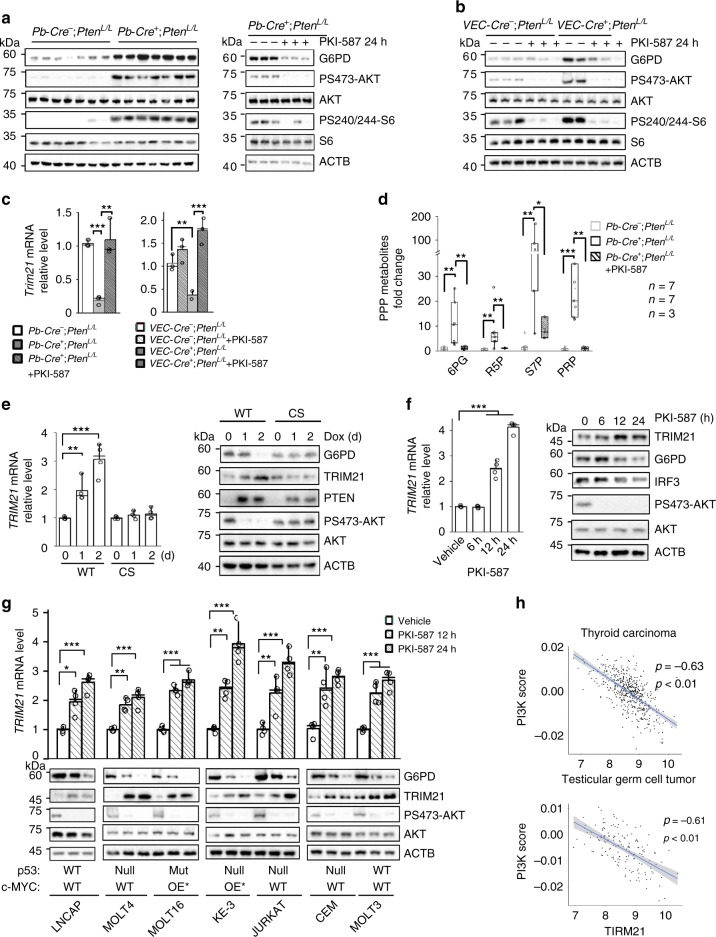
PI3K/AKT regulates TRIM21 and PPP in vivo and in human cancers. **a**–**d** *Pten* null prostate cancer and T-ALL mouse models have higher levels of G6PD protein and PPP metabolites as well as lower levels of *Trim21*, which could be reverted by PKI-587 treatment. The cell lysates from the anterior lobes of the prostates of *Pb-Cre*^−^*;Pten*^*L/L*^ and *Pb-Cre*^*+*^*;Pten*^*L/L*^ models (**a**) and the thymic cells of *VEC*^−^*Cre*^−^*;Pten*^*L/L*^ and *VEC*^*−*^*Cre*^*+*^*;Pten*^*L/L*^ mice (**b**) without and with PKI-587 (25 mg/kg) treatment; the *Trim21* mRNA levels (**c**) and PPP metabolites were measured (**d**) 24 h later. Median and quartile values are provided by the central line and box boundaries. Whiskers show min to max values. **e** *PTEN* WT/CS-inducible PC3 cells were treated with Dox (2 μg/ml) for the indicated time periods. The *TRIM21* mRNA and protein levels were measured by RT-qPCR, and cell lysates were subjected to immunoblotting with the indicated antibodies. **f** PC3 cells were treated with PKI-587 (1 µM) for the indicated time periods. The *TRIM21* mRNA levels were measured by RT-qPCR, and cell lysates were subjected to immunoblotting with the indicated antibodies. **g** Human T-ALL and LNCAP prostate cancer cells were treated with PKI-587 (1 µM) for the indicated time periods. The *TRIM21* mRNA and protein levels were measured by RT-qPCR, and cell lysates were subjected to immunoblotting with the indicated antibodies. **h** Correlations between the PI3K pathway activity score and the *TRIM21* mRNA levels in human thyroid and testicular cancers. Data are presented as fold changes and the mean ± SD and were compared with WT or untreated cells. Each experiment was performed *n* = 3 (**c**) and *n* = 4 (**e**–**g**) independent times. *p < 0.05, **p < 0.001, and ***p < 0.001, based on Student’s *t* test (two-sided ANOVA). See also Supplementary Fig. 4. Source data are provided as a Source Data file.

Similar to our findings in the isogenic mES cell lines, we observed significantly higher levels of total G6PD protein and lower levels of *Trim21* mRNA in the *Pten* null T-ALL and prostate cancer models compared with their WT littermates (Fig. 4a–c). Importantly, PKI-587 treatment could reduce the G6PD protein levels, accompanied by increased *Trim21* expression and decreased PPP metabolites (Fig. 4a–d; Supplementary Fig. 4A), providing strong in vivo support for this PI3K-regulated mechanism.

PI3K/AKT signaling also plays a similar role in regulating TRIM21 expression and G6PD protein levels in human cancers. The *TRIM21* mRNA and protein levels were upregulated when the WT PTEN, but not the phosphatase-dead CS PTEN, was re-expressed in the PTEN null prostate cancer PC3 cells, accompanied by decreased G6PD levels (Fig. 4e). Overexpression of exogenous *TRIM21* in PC3 cells blocked the effect of PI3K and reduced the G6PD protein levels (Supplementary Fig. 4B), supporting the notion that TRIM21 functions downstream of the PI3K pathway in regulating G6PD degradation. PKI-587 treatment of PC3 cells also led to increased *TRIM21* mRNA and protein levels, accompanied by reduced G6PD and decreased protein levels of the known TRIM21 substrate IRF3^30^ in a time-dependent manner (Fig. 4f).

We further extended our studies by analyzing additional prostate cancer and T-ALL lines, all of which carried *PTEN* mutations or deletions^31,32^ (Supplementary Table 2). Since the tumor suppressor p53 and oncogene c-MYC play critical roles in regulating glucose metabolism^5,26,27^, we also included cancer lines with p53 mutations or MYC overexpression to test whether the PI3K-regulated *TRIM21* expression depends on p53 or MYC status. All human cancer cells tested responded to PKI-587 treatment in a similar manner, i.e., time-dependent increases in the *TRIM21* mRNA and protein levels, accompanied by decreased total G6PD levels, which are independent of p53 and MYC status (Fig. 4g). Importantly, a strong negative correlation was also found between PI3K pathway activity and *TRIM21* expression in several human cancers (Fig. 4h; Spearman’s rank correlation rho was −0.63 and −0.61, respectively, with a *p* value lower than 0.01; Supplementary Fig. 4C). Taken together, these data suggested that the PI3K/AKT pathway negatively regulates the expression of the E3 ligase *TRIM21*, which in turn controls the level and activity of the PPP rate-limiting enzyme G6PD via ubiquitin-mediated degradation in vivo and in human cancers.

### PPP activates AKT and supports cell growth

**Fig. 5:**
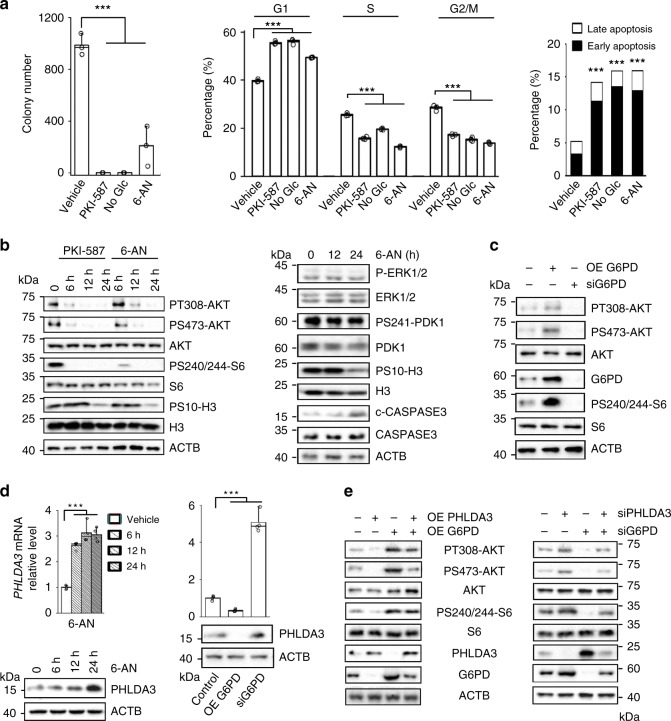
PPP promotes cell growth and AKT activation by inhibiting PHLDA3. **a** 6-AN treatment inhibits cell growth, similar to glucose starvation and PKI-587 treatment. *Pten* null mES cells were seeded in 3.5 cm dishes at a density of 2000 cells/well. Forty-eight hours later, the cells were treated with PKI-587 (1 µM), 6-AN (100 nM) or glucose depletion. The colony numbers were counted 7 days later (left panel), while the percentages of cells in each phase of the cell cycle and apoptosis were measured by FACS analysis after 24 h of PKI-587 (1 µM) or 6-AN (100 nM) treatment or 12 h in glucose-depleted medium (right panel, lower panel). **b** 6-AN treatment inhibits AKT activity, similar to the effect of PKI-587 treatment (left panel), but has no effect on the ERK and PDK1 activities (right panel). The *Pten* null mES cells were treated with PKI-587 (1 µM) or 6-AN (100 nM) for the indicated time periods. Cell lysates were subjected to immunoblotting with the indicated antibodies. **c** Overexpression or knockdown of G6PD alters the AKT activities in PC3 cells. **d** The *PHLDA3* mRNA and protein levels are regulated by 6-AN and *G6PD* activity in PC3 cells. **e** The PPP regulates AKT activity through a PHLDA3-dependent mechanism. *PHLDA3* overexpression abolished the *G6PD* overexpression-induced AKT activation (left), while *PHLDA3* knockdown blocked the *G6PD* knockdown-induced AKT inactivation in PC3 cells (right). Data are presented as the mean ± SD and were compared with untreated cells. Each experiment was performed *n* = 3 (**a**) and *n* = 4 (**d**) independent times. *p < 0.05, **p < 0.001, and ***p < 0.001, based on Student’s *t* test (two-sided ANOVA). See also Supplementary Fig. 5. Source data are provided as a Source Data file.

While most recent studies have focused on how oncogenic pathways reprogram metabolic processes to meet the needs of uncontrollable cell proliferation, whether altered cell metabolism also provides feedback to oncogenic pathways to reinforce the vicious cycle of tumor growth is unclear. We found that the PPP plays an essential role in cell growth even in the presence of a constitutively activated PI3K pathway in the PTEN null cells. Blocking the PPP by 6-AN significantly inhibited mES colony formation in the presence of a normal concentration of glucose, similar to the effects of glucose starvation and PKI-587 treatment (Fig. 5a, left panel). FACS analysis further demonstrated that the aforementioned treatments led to similar levels of cell cycle blockage as well as apoptosis (Fig. 5a, middle and right panels). Since PI3K/AKT plays a central role in cell proliferation and survival, we hypothesized that the PPP may feedback regulate PI3K/AKT activity.

Indeed, 6-AN treatment effectively inhibited AKT phosphorylation and activation of its downstream effector S6, similar to PKI-587 treatment (Fig. 5b, left panel). Importantly, in association with the 6-AN- or PKI-587-mediated AKT inhibition, the phospho-H3 levels were significantly decreased, while the c-CASPASE3 levels were significantly increased (Fig. 5b). However, 6-AN treatment had no effect on the ERK and PDK1 activities, suggesting an AKT-specific effect (Fig. 5b, right panel).

We also tested the association between the PPP and AKT activity in the *PTEN* null human prostate cancer and T-ALL cell lines. We found that 6-AN treatment substantially decreased the P-AKT levels, similar to the effects of PKI-587 treatment or glucose starvation, accompanied by significant changes in apoptosis and the cell cycle (Supplementary Fig. 5A, B). Therefore, the PPP also activates AKT and supports cell growth and survival in human cancer cells.

To avoid nonspecific off-target effects of 6-AN, we manipulated the PPP activity by overexpressing or knocking down its rate-limiting enzyme G6PD in the *PTEN* null prostate cancer PC3 cells. G6PD overexpression increased AKT phosphorylation, while *G6PD* knockdown significantly decreased AKT phosphorylation (Fig. 5c). *G6PD* knockdown also significantly altered the cell cycle and enhanced apoptosis, accompanied by decreased colony number and cell viability without affecting ERK phosphorylation (Supplementary Fig. 5C–E), similar to 6-AN treatment. Thus, increased PPP metabolism can activate AKT through a positive feedback mechanism even in *PTEN* mutant cells and in the presence of the constitutively activated PI3K pathway.

### PPP controls AKT activation by inhibiting PHLDA3

To explore the mechanism of PPP-regulated AKT activation, we conducted RNAseq analysis of the 6-AN-treated cells. We found that among those molecules known to negatively regulate AKT activation, the pleckstrin homology-like domain family A member 3 (*PHLDA3)* mRNA and protein levels were significantly increased following 6-AN treatment (Fig. 5d, left panel). Similarly, the *PHLDA3* mRNA and protein levels were increased upon *G6PD* knockdown and decreased upon *G6PD* overexpression in the human PTEN null PC3 prostate cancer cells (Fig. 5d, right panel), indicating that *PHLDA3* expression is negatively regulated by the PPP.

PHLDA3 suppresses AKT activation by directly competing with the AKT PH domain for binding to the membrane-associated PIP3, the first step in AKT activation^33^, which may explain the AKT-specific effect observed in our study (Fig. 5b). We therefore further determined the functional relevance of PHLDA3 in the PPP-regulated AKT activation in the *G6PD*-overexpressing or *G6PD*-knockdown cells. AKT activation was significantly abolished by *PHLDA3* overexpression even in the *G6PD*-overexpressing cells (Fig. 5e, left panel). *PHLDA3* knockdown, however, led to increased AKT activation and resistance to *G6PD*-knockdown- or 6-AN-mediated AKT inhibition (Fig. 5e, right panel; Supplementary Fig. 5F). Together, these results indicate that PHLDA3 acts downstream of the G6PD-regulated PPP and that PPP feedback regulates AKT activation by inhibiting the expression of *PHLDA3*.

### PPP metabolites promote AKT activation by inhibiting PHLDA3

**Fig. 6:**
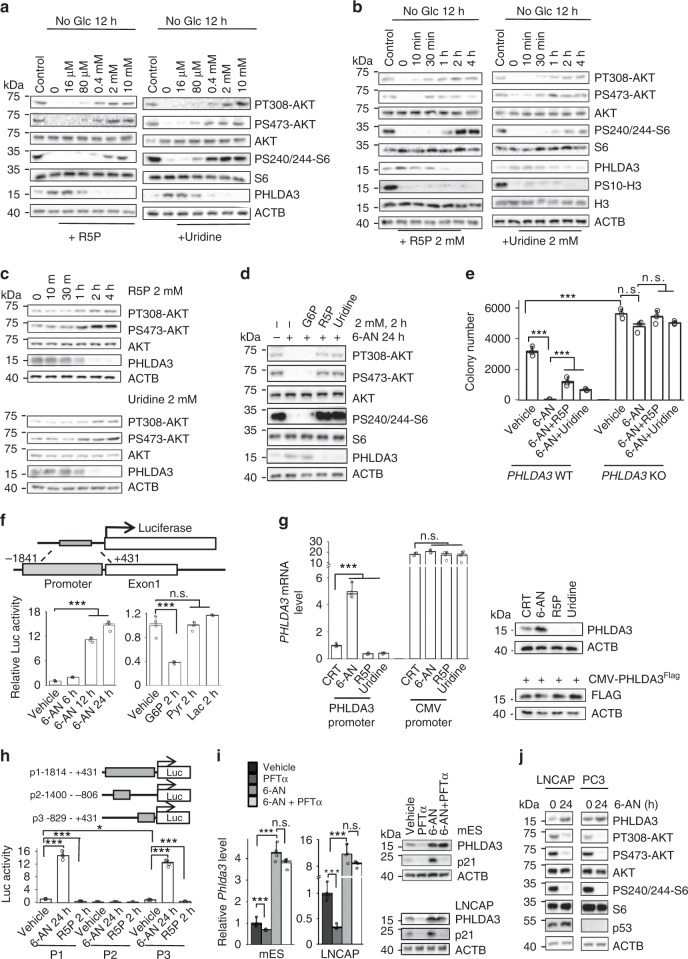
PPP metabolites promote AKT activation by inhibiting PHLDA3. **a**–**c** R5P or uridine blocks glucose starvation-induced PHLDA3 upregulation and AKT inhibition. *Pten* null mES cells were treated with various concentrations of R5P or uridine for 4 h (**a**), or indicated time periods with (**b**) with or without (**c**) 12 h of glucose starvation. Cell lysates were subjected to immunoblotting with the indicated antibodies. **d** The *Pten* null mES cells were treated with 6-AN for 24 h, followed by treatment with R5P, uridine and G6P for 2 h. Cell lysates were subjected to immunoblotting with the indicated antibodies. **e** *PHLDA3* WT and knockout HeLa cells were treated with 6-AN in the presence or absence of R5P or uridine. Three days later, colony numbers were counted. **f** PPP controls PHLDA3 expression by regulating its promoter activity. PC3 cells transfected with the PHLDA3-luciferase reporter plasmid (upper) were treated with 6-AN or incubated with PPP metabolites for 2 h. The cell lysates were harvested for the luciferase activity assay (lower). **g** PHLDA3 driven by an exogenous promoter fails to respond to 6-AN treatment or R5P and uridine supplements. PC3 cells were transfected without or with a CMV-driven FLAG-PHLDA3 expression plasmid and treated with 6-AN for 24 h or R5P or uridine for 2 h. Cell lysates were subjected to RT-qPCR or immunoblotting with the indicated antibodies. **h** Truncation analysis narrows down the *PHLDA3* promoter region necessary for PPP regulation to p3 (−829 to +431). **i, j** PPP regulates PHLDA3 and AKT independent of p53 status. *Pten* null mES cells and LNCAP cells (*p53* WT) were treated without or with PFTα (5 μM) or 6-AN (100 nM). The *Phlda3* mRNA and protein levels were measured (**i**). *PTEN* null LNCAP (*p53* WT) and PC (*p53* null) cells were treated with 6-AN. Cell lysates were subjected to immunoblotting with the indicated antibodies (**j**). Data are presented as the mean ± SD and were compared with untreated cells. Each experiment was performed *n* = 3 (**e**) and *n* = 4 (**f**–**i**) independent times. *p < 0.05, **p < 0.001, and ***p < 0.001, based on Student’s *t* test (two-sided ANOVA). See also Supplementary Fig. 5. Source data are provided as a Source Data file.

To further investigate how the PPP regulates AKT activation, we tested several key PPP metabolites that could be taken up by cells from the media^34,35^. The addition of R5P or uridine effectively rescued the glucose starvation-induced P-AKT reduction in a concentration-dependent manner, accompanied by decreased PHLDA3 levels (Fig. 6a). We then conducted a time-course study and found that exogenous R5P and uridine could incorporate into the PPP within 10 min (Supplementary Fig. 6A), and significantly downregulate the PHLDA3 mRNA and protein levels and activate AKT within 30–60 min with or without glucose starvation (Fig. 6b, c; Supplementary Fig. 6B). These findings suggested that the PPP-PHLDA3-AKT feedback loop functions under both normal and glucose-starved conditions. The effects of R5P and uridine on AKT activation are probably not due to increased cell proliferation or inhibition of the PPP, as the H3-S10 phosphorylation and PPP intermediate metabolite levels did not change significantly within the time window tested (Fig. 6b; Supplementary Fig. 6A). P-ERK and P-PDK1 showed no obvious changes after R5P or uridine addition, further confirming that PPP-regulated cell signaling is AKT-specific (Supplementary Fig. 6C).

Since parts of glycolysis and the PPP are interconvertible, we further tested the requirement of the PPP for AKT activation by adding glycolytic metabolites that can or cannot convert to PPP intermediates. The PPP upstream metabolite G6P could activate AKT, while the glycolytic downstream products pyruvate and lactate, which cannot convert to PPP metabolites, did not significantly affect the P-AKT and PHLDA3 levels within the time window tested (Supplementary Fig. 6D). In addition, treating cells with the PPP inhibitor 6-AN or the glycolysis inhibitor 2-DG had no effect on R5P or uridine-induced PHLAD3 downregulation and AKT activation, but 6-AN could block the G6P-induced AKT activation (Fig. 6d; Supplementary Fig. 6E), suggesting a PPP-specific regulatory mechanism. Notably, the concentrations of R5P and uridine were decreased below physiological levels after 6-AN treatment or glucose starvation, while R5P or uridine supplementation could rescue their levels within 10 min (Supplementary Fig. 6F), indicating that the concentrations of PPP metabolites are important for the PHLDA3-regulated AKT inhibition.

The 6-AN-induced growth inhibition was PHLDA3-dependent, and clonogenic growth inhibition caused by 6-AN treatment could be partially rescued by R5P or uridine only in the *PHLDA3* WT cells (Fig. 6e). Therefore, PHLDA3 acts downstream of the PPP and negatively regulates AKT activity and cell proliferation.

### PPP metabolites inhibit *PHLDA3* expression independent of p53

To further investigate the mechanism underlying PPP-regulated *PHLDA3* transcription, we constructed a *PHLDA3-luciferase* reporter construct containing the −1841 to +431 region of the human *PHLDA3* gene (Fig. 6f, upper panel). When transfected into PC3 cells, the reporter construct showed similar kinetics of response to 6-AN treatment and R5P or uridine supplementation compared with the endogenous gene (Fig. 6f, lower left panel; Supplementary Fig. 6G). G6P showed similar inhibition, while pyruvate and lactate had no obvious effects on the PHLDA3 promoter activity (Fig. 6f, lower right panel). Importantly, *PHLDA3* driven by an exogenous promoter did not respond to 6-AN treatment or R5P and uridine supplementation (Fig. 6g).

Truncation analysis further identified the minimal region for PPP inhibition as −829 to +431 (Fig. 6h). *PHLDA3* expression is known to be regulated by the tumor suppressor p53^33,36^. The region in which p53 binds to the *PHLDA3* promoter overlaps with the minimal region required for PPP-regulated *PHLDA3* expression, which prompted us to investigate whether the PPP-regulated *PHLDA3* expression is p53-dependent. For this, we tested the effect of the p53 inhibitor PFTα on the 6-AN-induced *PHLDA3* expression. Although we observed reduced *Phlda3* levels when we treated the *Pten* null mES cells and LNCAP cells with the p53 inhibitor PFTα, we did not observe any significant difference in the 6-AN treatment-induced *Phlda3* expression without or with PFTα (Fig. 6i). Importantly, *PTEN* null *p53* WT LNCAP and *PTEN* and *p53* null PC3 human prostate cancer lines showed similar responses to 6-AN treatment (Fig. 6j). These results suggest that the PPP-regulated *PHLDA3* expression is p53-independent.

### PPP controls cell proliferation and survival in vivo

**Fig. 7:**
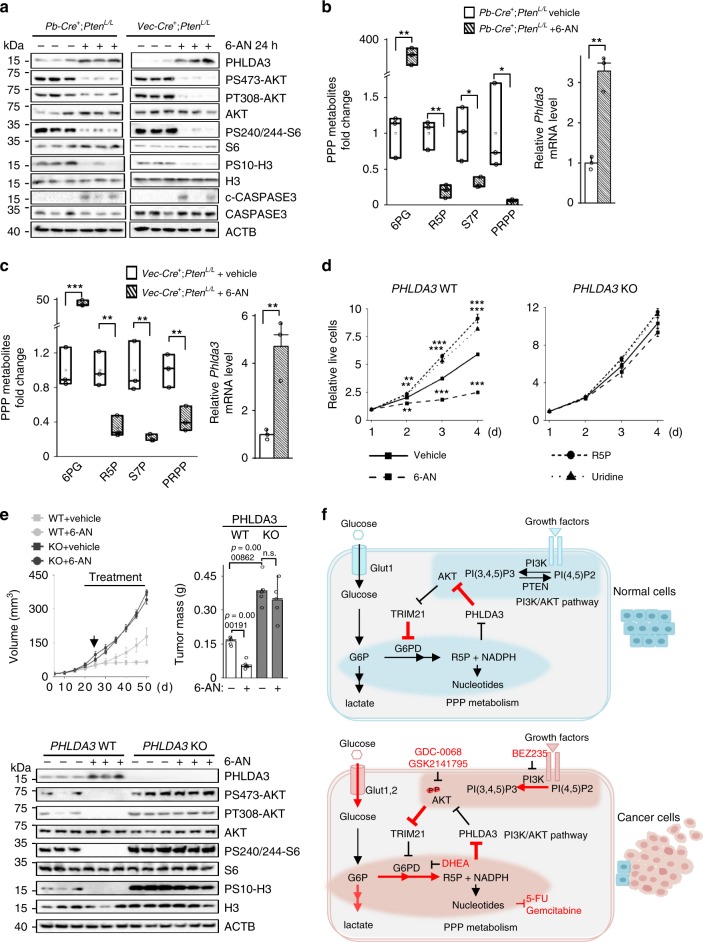
PPP controls cell proliferation and survival in vivo. **a**–**c** The effects of PPP inhibition in the *Pten* null prostate cancer and T-ALL mouse models. Mice were treated with of 6-AN (15 mg/kg) for 24 h. Metabolites were measured by LC-MS (left panel). mRNA and proteins were detected by RT-PCR or immunoblot. Median and quartile values are provided by the central line and box boundaries. Whiskers show min to max values. **d** PHLDA3 knockout blocks the effects of 6-AN, R5P or uridine on cell growth in vitro. Isogenic *PHLDA3* WT and knockout HeLa cells were seeded in 96-well plates at a density of 1000 cells/well. After 48 h, the cells were treated with 6-AN (100 nM) or 2 mM R5P or uridine. Cell viability was detected by CCK-8 assays at indicated time points. **e** *PHLDA3* knockout blocks 6-AN-induced cell growth in vivo. Equal numbers of isogenic *PHLDA3* WT and *PHLDA3* knockout HeLa cells were implanted onto the bilateral flanks of nude mice. Tumor volumes and weights were measured and presented (upper) and cell lysates from the xenograft tumors were subjected to immunoblotting with the indicated antibodies (lower). **f** Schematic representation of the crosstalk between the PI3K/AKT pathway and PPP metabolism. TRIM21 and PHLDA3 are two negative regulators identified in this study. TRIM21 and PHLDA3 play essential roles in regulating the AKT-TRIM21-G6PD (PPP) and PPP-PHLDA3-AKT feedback loops, respectively, in this signaling pathway-metabolic pathway crosstalk. Targeting the positive regulators of these crosstalk mechanisms, as indicated in the lower panel, may be an effective treatment for cancers with PTEN loss or PI3K activation. Loss of the negative regulators of these crosstalk mechanisms may contribute to tumor development and treatment resistance. Data are presented as the mean ± SD and were compared with WT or vehicle-treated cells. **a**–**c**: *n* = 3, number of independent mouse samples; **d**: *n* = 3 independent experiments; **e**: *n* = 5 xenografts. *p < 0.05, **p < 0.001, and ***p < 0.001, based on Student’s *t* test (two-sided ANOVA). See also Supplementary Fig. 7. Source data are provided as a Source Data file. The PI3K/AKT signaling pathway regulates cancer metabolism. Here, the authors show a reciprocal positive feedback crosstalk between the PI3K/AKT and pentose phosphate pathway which promotes tumourigenesis.

As a very small percentage of glycolytic intermediates are shunted through the PPP in normal cells, the dependence of PTEN null cells on the PPP may represent an attractive therapeutic opportunity. To test this hypothesis, we treated the *Pten* null prostate cancer and T-ALL models with 6-AN and observed significantly increased the *Phlda3* mRNA and protein levels, accompanied by substantial decreases in the levels of P-AKT and P-S6 as well as the PPP metabolic intermediates (except for 6PG since 6-AN blocks the first two steps of the PPP) (Fig. 7a–c). As a consequence of the decreased AKT activity, cell proliferation was inhibited, as evidenced by the decreased phospho-H3 levels, while cell death was increased, as evidenced by the increased cleaved CASPASE3 levels (Fig. 7a). These results indicated that the PTEN null cancer cells are sensitive to anti-PPP therapies even in the presence of a hyperactivated PI3K pathway.

The response of the cells to PPP inhibition is PHLDA3-dependent. *PHLDA3* knockout cells had hyperactivated AKT and did not respond to the inhibitory effects of 6-AN on AKT phosphorylation and cell growth (Fig. 7d; Supplementary Fig. 7A). As PHLDA3 acts downstream of PPP, the *PHLDA3* knockout cells did not respond to the growth promotion effect of R5P or uridine supplementation compared with the *PHLDA3* WT cells (Fig. 7d). We also tested the effects of 6-AN treatment on the *PHLDA3* WT- and knockout-derived xenograft models in vivo. As shown in Fig. 7e, 6-AN treatment could not inhibit *PHLDA3* knockout xenograft tumor growth in vivo (Fig. 7e, upper panels; Supplementary Fig. 7B), most likely due to the constitutively activated AKT pathway (Fig. 7e, lower panel). These results suggest that cancers with *PHLDA3* loss may be resistant to anti-PPP inhibitor treatment.

## Discussion

Most cancer metabolic studies are focused on how oncogenes and tumor suppressor genes, as well as their controlled signaling pathways, regulate cell metabolism. Our study suggests that the metabolic pathway is not a passive recipient of oncogenic signals; rather it feedback regulates oncogenic signals. In normal cells at steady state, PTEN restrains PI3K/AKT activity and promotes TRIM21-mediated G6PD degradation, consequently lowering PPP activity and cell proliferation. PPP metabolites, on the other hand, maintain AKT activity by negatively regulating the expression of its inhibitor *PHLDA3*. Loss of the PTEN tumor suppressor or activation of the PI3K/AKT pathway in cancer cells enhances G6PD activity and the PPP by inhibiting TRIM21. Increased PPP metabolites, in turn, block *PHLDA3* expression and reinforce AKT activation. The reciprocal crosstalk between oncogenic signaling and metabolic pathways, therefore, may serve as a positive enforcement for cancer metabolic reprogramming and ensure uncontrollable cancer growth. Cotargeting the positive regulators of these reciprocal crosstalk mechanisms, such as PI3K, AKT, G6PD and nucleotide synthesis^37–42^ may be an effective treatment for cancers with PTEN loss or PI3K activation. The negative regulators in these crosstalk mechanisms, such as TRIM21 and PHLDA3 discovered in this study, may contribute to cancer development and treatment resistance (Fig. 7f).

PTEN loss or PI3K activation not only promotes the Warburg effect by increasing the level of a glucose transporter and the rate of glycolysis, but also decouples glycolysis and the TCA cycle and diverts glycolytic intermediates to the PPP branching metabolic pathway (Fig. 1). The effect of PI3K/AKT activation on the PPP observed in this study differs from that of the oncogene KRAS^43^. While both the oxidative and nonoxidative arms of the PPP, together with PPP-associated nucleotides and NADPH production, were upregulated in *Pten* null cells (Fig. 1), only the nonoxidative arm was upregulated in a *Kras* pancreatic cancer model^43^. These differences are most likely mediated by the mechanisms by which PI3K/AKT and KRAS control the PPP. PI3K/AKT controls the activity of G6PD, which regulates the oxidative arms of the PPP and determines the speed of glycolytic intermediate flux into the PPP (Fig. 2). AKT can also modulate TKT activity, a key enzyme in the nonoxidative PPP^44,45^. KRAS, however, controls the expression of enzymes involved in the nonoxidative arm of the PPP^43^. As the oxidative arm of the PPP is irreversible and important for nucleotide and NADPH production^45^, the difference between the effects of PI3K/AKT and KRAS signaling on the PPP may explain the increased oxidative stress associated with KRAS tumors.

Ubiquitylation and proteasome-mediated degradation are instrumental in regulating protein functions. In our study, PTEN loss or PI3K/AKT activation prolonged the G6PD half-life by preventing its ubiquitin-mediated degradation (Fig. 2). We also identified TRIM21 as the E3 ligase for G6PD, which is transcriptionally downregulated by PI3K/AKT activation (Figs. 3 and 4). Our analysis demonstrated that the *TRIM21* expression levels are negatively correlated with PI3K activity in various human cancers, implying that TRIM21 may act as a potential tumor suppressor. How the PI3K pathway regulates *TRIM21* expression requires further study. Although AKT downstream substrates, such as FOXO3A, could play a role in the PI3K/AKT/mTORC2-regulated *TRIM21* expression, we did not find a FOXO3A binding sequence in the *TRIM21* promoter. We have conducted unbiased bioinformatics analyses on the *TRIM21* promoter using the consensus binding sequences of major transcription factors^46^ and will conduct functional tests on those potential candidates. TRIM21 can also promote phosphofructokinase 1 (PFK1) degradation^29^. Therefore, TRIM21 may act at multiple nodes to control normal cell metabolism and cancer metabolic reprogramming.

We also discovered a pathway by which PPP metabolite feedback positively regulates AKT activation, even in the presence of a constitutively activated PI3K pathway (Figs. 5–7). PPP metabolites, such as R5P and uridine, can promote AKT activation, cell proliferation and survival by inhibiting the tumor suppressor PHLDA3 (Figs. 5 and 6). *PHLDA3* can negatively regulate AKT activation^33^, and *PHLDA3* loss of heterozygosity has been reported in human lung and neuroendocrine pancreatic cancers^33,36^. We found that in the *Pten* null mES and in vivo cancer models, the abundant PPP metabolites can restrain *PHLDA3* expression, which in turn activates AKT and further promotes cell growth. Importantly, a similar control mechanism was also present in the human prostate and T-ALL lines.

How PPP metabolites regulate *PHLDA3* expression is currently unknown, but recent studies have demonstrated several possible underlying mechanisms. Cell metabolites can either function as signaling molecules or indirectly modulate metabolic sensors or transcription factors to control transcriptional regulation, epigenetic remodeling, cell differentiation and cancer development. During mammalian zygotic genome activation, pyruvate can trigger TCA cycle-associated enzymes to translocate to the nucleus and regulate histone modification and global epigenetic changes^47^. Intracellular nutrients can regulate the activities of the nutrient and energy sensors mTORC1^48–50^ and AMPK^51,52^. The oncometabolite 2-HG, generated by mutated IDH1/2, can also regulate the epigenetic program and influence cancer development^53,54^. Although we have not found the sensor responsible for PPP-regulated *PHLDA3* expression, our study indicates that such regulation is p53-independent and is not mediated by shutting down the PPP. PPP-regulated cell signaling is also AKT-specific, as it has no effect on the activities of either its upstream factor PDK-1 or its parallel pathway ERK (Fig. 6). Therefore, our study discovered a reciprocal feedback mechanism between the PI3K/AKT pathway and PPP metabolism (Fig. 7f), which not only orchestrates the partitioning of glycolytic intermediates to branching metabolic pathways to meet the needs of rapid tumor cell growth but also reinforces PI3K/AKT activation and aerobic glycolysis for cancer metabolic reprogramming.

As a very small percentage of glucose is shunted through the PPP in normal cells, the dependency of PTEN null cells on the PPP and the reciprocal crosstalk between the PI3K/AKT pathway and the PPP may represent an attractive therapeutic opportunity. As a proof of principle study, we revealed that PPP inhibition impeded AKT activation and the proliferation and survival of PTEN null mES cells and human cancer cells, as well as in vivo cancer models, indicating that PTEN null cancer cells may be vulnerable to anti-PPP therapy, even in the presence of a hyperactivated PI3K pathway. We also demonstrated that the effects of PPP inhibition on AKT activation and cell proliferation are PHLDA3-dependent and predict that the PHLDA3 status in human cancers may dictate their response to anti-PPP treatments. Although documented PHLDA3 KO cases are limited, we did find the association between low PHLDA3 protein levels and poor cancer-specific and disease-free survival in 26% esophageal squamous cell carcinoma patients^55^. However, PTEN and PI3K/AKT status cannot be tracked from this study, nor from other PHLDA3-associated human cancer studies^33,36^. Therefore, it is difficult at this moment to predict the percentages of cancer patients with *PTEN* null/*PHLDA3* vs. *PTEN* null/*PHLDA3* KO genotypes. DHEA is a G6PD inhibitor that has been used in clinic although the in vivo anti-G6PD effect is subject to debate^56^, Many drugs targeting downstream of PPP, such as 5-Fluorouracil and Gemcitabine, have been approved for anticancer treatment^42,57^. Further works are needed for more specific and effective anti-PPP therapies.

## Methods

### Antibodies and chemical compounds

Antibodies and chemical compounds used in this study are listed in Supplementary Table 3.

### Cell lines

*Pten* WT, null and mutant (CS and GE) mES cells were established by our lab and adapted to feeder-free culture conditions and maintained in Knock-Out DMEM (Gibco) supplemented with 15% fetal bovine serum (HyClone), 1000 U/ml leukemia inhibitory factor (LIF), 2 mM L-glutamine, 100 U/ml penicillin/streptomycin, 0.1 mM nonessential amino acids (NEAA, Gibco), and 100 µM β-mercaptoethanol. Media were replaced every day and cells were passaged every 3 days. All experiments were performed on mES cells within 15 passages. Glucose-depleted medium for mES cells was purchased from Gibco. All human T-ALL, prostate cancer cell lines and HEK293T cells were purchased from ATCC and maintained accordingly. PC3 WT/CS-PTEN-inducible cells were established by our lab. Glucose-depleted media were purchased from Gibco. Isogenic *TRIM21-WT and* -knockout A549 cells, *PHLDA3*-WT and -knockout HeLa cells were generously provided by Dr. Wensheng Wei of Peking University and cultured in DMEM containing 10% fetal bovine serum and 100U/ml penicillin/streptomycin.

### Mice

The generation of the *Pb-Cre*^+^*;Pten*^*loxP/loxP*^ prostate cancer model and *VEC-Cre*^*+*^;*Pten*^*loxP/loxP*^ T-ALL model has been described previously^19,21^. Male CAnN.Cg-Foxn1nu/CrlVr mice were purchased from Charles River Laboratories China. Mouse genotypes were verified by PCR with primers for *Pten* and *Cre*^21^. Prostate and thymic tissues were isolated for metabolite measurements, Western blotting and mRNA analysis. Animal housing, breeding, and surgical procedures were approved by the Ethics Committee under ID LSC-WuH-1 and conducted in accordance with the regulations of the Division of Laboratory Animal Medicine at Peking University.

### Plasmids and transfection

The expression vectors of 3×Flag-G6PD, Flag-TRIM21, GST-TRIM21, and the TRIM21 mutants were kindly provided by Prof. Peng Jiang (Tsinghua University). The HA-ubiquitin expression vector was kindly provided by Prof. Xin Ye (Institute of Microbiology, CAS). The Flag-PHLDA3 vectors were constructed by cloning PHLDA3 cDNA into the pFLAG-CMV-2 vectors (Sigma). The Flag-G6PD WT and Flag-G6PD K8R vectors were constructed by cloning G6PD WT or mutant (K8R) cDNA into the pFLAG-CMV-2 vectors. Myc-PTEN was generated by cloning PTEN cDNA into the pCMV myc vector (Clontech). The expression plasmid of AKT WT was kindly provided by Prof. Peng Jiang. The expression plasmids of AKT mutants were generated from AKT WT plasmid. The PHLDA3 promoter was cloned into the pGl3.1 plasmid to drive the luciferase reporter assay. The following primer pairs were used for PHLDA3 promoter cloning: promoter −1841 to +431 (forward):

5’- CCGCTCGAGTTAGCCAGGTGTGGTGGCAGATGCCTG - 3’

promoter −1841 to +431 (reverse):

5’- CCCAAGCTTCCAGCACGCCCTCCTTGAGCACGGTA - 3’

promoter −1400 to −806 (forward):

5’- CCGCTCGAGTTAGCCAGGTGTGGTGGCAGATGCCTG - 3’

promoter −1400 to −806 (reverse):

5’- CCCAAGCTTCCAGCACGCCCTCCTTGAGCACGGTA - 3’

promoter −829 to +431 (forward):

5’- CCGCTCGAGTTAGCCAGGTGTGGTGGCAGATGCCTG - 3’

promoter −829 to +431 (reverse):

5’- CCCAAGCTTCCAGCACGCCCTCCTTGAGCACGGTA - 3’

PC3 or HEK293T cells were transfected with constructs for 36–48 h using Lipofectamine 2000 (Invitrogen).

### Glucose consumption and lactate production

mES cells were cultured for 24 h and then changed into fresh ES cell culture medium. Eight hours later, the glucose levels in the culture medium were measured using the Glucose (GO) Assay Kit (Sigma), and the lactate levels were determined using a Lactate Assay Kit (Eton Bioscience).

### ECAR assay

WT and *Pten* mutant mES cells were seeded in 24-well plates at a density of 2 × 10^4^ cells/well and cultured under standard ES culture conditions for 12 h. The cells were then cultured in glucose-depleted media for 1 h, and the extracellular acidification rate (ECAR) was measured after the addition of glucose (80 mM), oligomycin (2 mM), and 2-DG (50 mM) using a Seahorse XF24 instrument (Seahorse Bioscience). The Seahorse XF Glycolysis Stress Test Kit was purchased from Agilent Technologies.

### RNA interference

PC3 cells were transfected with gene-specific small interfering RNAs (siRNA) or a control siRNA (50 nM) for 72 h using Lipofectamine 2000. G6PD siRNAs were purchased from Invitrogen (Catalog No. HSS103891). PHLDA3 and TRIM21 siRNAs were purchased from RiboBio:

siPHLDA3: GGCCCAAGGAGCTCAGCTT

siTRIM21-#1: GGACAATTTGGTTGTGGAA

siTRIM21-#2: GGAATGCATCTCTCAGGTT

### Metabolite extraction and mass spectrometry-based metabolomics analysis

Analyses of glycolysis/PPP tracing were performed using standard procedures^23^. Briefly, cells were cultured in standard medium and then changed to medium containing 25 mM U-^13^C or 1,2-^13^C_2_ glucose for 12 h. Metabolites were extracted with cold 0.18 M aqueous trichloroacetic acid. Insoluble materials in lysates were centrifuged at 12,000 × *g* for 15 min, and the resulting supernatants were freeze-dried. Samples were resuspended in 30 µl of ultrapure water for liquid chromatography-high resolution mass spectrometry^58^. The concentrations of the extracted metabolites were normalized to the protein concentration measured using the BCA assay. Compounds were separated on an XBridge Amide column (100 × 4.6 mm, 3.5 μm; Waters, Milford, MA, USA). Buffer A comprised 20 mM ammonium acetate (pH 9.4) in water, and buffer B was acetonitrile (LC-MS grade). All standard metabolites were purchased from Sigma-Aldrich. The retention times of ATP and GTP were very close, and the concentration of GTP was close to the detection limit. Hence, we detected GDP instead of GTP.

### Coimmunoprecipitation assays

Cells were harvested and lysed in lysis buffer at 4 °C for 20 min. Cell lysates were incubated with Protein A-agarose at 4 °C for 2 h followed by the addition of antibodies overnight or Flag antibody-conjugated agarose beads for 4 h at 4 °C. The beads were then washed with lysis buffer. The immunoprecipitants were eluted from the beads with 2×SDS loading buffer, separated on 10% SDS-PAGE gels, and immunoblotted with the indicated antibodies.

### Enzymatic activity measurements

The pyruvate kinase activity assay was performed using a pyruvate kinase activity assay kit (Abcam, catalog number ab83432) according to the manufacturer’s protocol. The G6PD enzyme activity was determined in two steps^59^. First, the combined activity of G6PD and 6-phosphogluconate dehydrogenase (6PGD), both of which produce NADPH, was measured by the rate of conversion of NADP^+^ to NADPH after adding their substrates, glucose-6-phosphate (G6P, 200 μM) and 6-phosphogluconate (6PG, 200 μM). Then the activity of 6PGD alone was measured by the conversion of NADPC to NADPH in the presence of its substrate 6PG (200 μM). G6PD activity was calculated as the difference of these two activities. Reactions were carried out in 50 mM Tris and 1 mM MgCl2, pH8.1. Enzyme activities were normalized on the basis of protein concentration.

### Luciferase assay

PC3 cells were transfected with luciferase reporter plasmids or control plasmid, pGL3.1, for 24 h. The Steady-Glo® Luciferase Assay System (Promega) was used to quantify luminescence from the transfected cells.

### Colony formation assay

WT or *Pten* null mES cells were seeded in 3.5 cm dishes at a density of 2000 cells/well. After 24 h, the cells were treated with different drugs. After 4 days of drug treatment, the cells were fixed with methanol for 30 min and stained with trypan blue for 30 min at room temperature, and then, the colonies with a diameter greater than 100 mm were counted.

### Cell viability assay

*TRIM21* WT and KO cells or *PHLDA3* WT and KO cells were seeded in 96-well plates. After 24 h, the *PHLDA3* WT and KO cells were treated with 6-AN, R5P (2 mM), or uridine (2 mM) for 4 days. CCK-8 reagent (Yeasen) was added to each well and incubated for 1 h according to the manufacturer’s protocol. The mean absorbance at 450 nm was measured.

### Permeabilization with SLO

G6P treatment was accompanied by SLO permeabilization. PC3 cells were permeabilized using a procedure similar to a previously described method^60^. Briefly, PC3 cells were washed three times with IC buffer (140 mM potassium glutamate, 20 mM HEPES, 5 mM MgCl_2_, 5 mM EGTA, and 5 mM NaCl, pH 7.2) and incubated in IC buffer containing 0.5 IU/ml SLO and 2 mM G6P for 10 min at 37 °C for permeabilization. Then, the cells were changed into normal medium for the indicated times.

### Cell apoptosis assay

The collected cells were subjected to apoptosis detection using flow cytometry on a BD Accuri C6 flow cytometer (BD Biosciences, San Jose, CA, USA) using the Annexin V and PI apoptosis detection kit (MultiSciences), according to the manufacturer’s protocol.

### Cell cycle assay

Cells were harvested and fixed with ethanol overnight at −20 °C before staining with propidium iodide. Flow cytometry was performed with a FACScan instrument.

### Xenograft tumor formation assays

*TRIM21* WT or isogenic *TRIM21* knockout cells (2.5 × 10^6^ cells) were mixed 1:1 in LDEV-free Matrigel (Corning, SLS 354234) and injected subcutaneously into the bilateral flanks of male CAnN.Cg-Foxn1^nu^/CrlVr mice. Xenograft tumors were measured twice every six days, and tumor volume was calculated using the following calculation: volume (mm^3^) = (length × width × width)/2.

For 6-AN treatment, an equal number of *PHLDA3* WT or isogenic *PHLDA3* knockout cells (2.5 × 10^6^ cells) were mixed 1:1 in Matrigel and implanted onto the bilateral flanks of nude mice. Xenograft growth was monitored daily. After the tumors became palpable, the mice were treated with 1 mg/kg 6-AN once daily IP for 30 days. Then, 6-AN was freshly prepared in 1% DMSO in PBS. Xenograft tumors were measured every six days with a caliper, and tumor volumes and weight were calculated as described above.

### Reverse transcription and PCR analysis

Total RNA and cDNA were prepared using TRIzol (Invitrogen) and qPCR (Vazyme R223-01). PCR was performed according to the manufacturer’s instructions (Vazyme Q121-03).

The following primer pairs were used for quantitative real-time PCR:

Human GAPDH (forward): 5’-GGGGAGCCAAAAGGGTCATCATCT-3’

Human GAPDH (reverse): 5’-GAGGGGCCATCCACAGTCTTCT-3’

Mus Actb (forward): 5’-GTGACGTTGACATCCGTAAAGA-3’

Mus Actb (reverse): 5’-GCCGGACTCATCGTACTCC-3’

Human PHLDA3 (forward): 5’-CCGTGGAGTGCGTGGAGAGC-3’

Human PHLDA3 (reverse): 5’-CTAGGGTGATCTGGGCGTTCC-3’

Mus Phlda3 (forward): 5’-CCGTGGAGTGCGTAGAGAG-3’

Mus Phlda3 (reverse): 5’-TCTGGATGGCCTGTTGATTCT-3’

Human TRIM21 (forward): 5’-TCAGCAGCACGCTTGACAAT-3’

Human TRIM21 (reverse): 5’-GGCCACACTCGATGCTCAC-3’

Mus Trim21 (forward): 5’-TGGTGGAGCCTATGAGTATCG-3’

Mus Trim21 (reverse): 5’-GGCACTCGGGACATGAACTG-3’

Mus G6pd (forward): 5’-CACAGTGGACGACATCCGAAA-3’

Mus G6pd (reverse): 5’-GCAGGGCATTCATGTGGCT-3’

### Western blot analysis

Cells were harvested, lysed in RIPA buffer (1% SDS) containing protease inhibitors (Roche Applied Science), and quantified using a BCA Protein Assay Kit (Pierce). Cell extracts were separated on 10% SDS-PAGE gels, transferred to PVDF membranes (Millipore), blocked with Tris-buffered saline/Tween 20 (TBST) containing 5% skim milk for 1 h at room temperature, and then probed with the indicated primary antibody at 4 °C overnight. After three washes, the membranes were incubated with a 1:5,000 dilution of HRP-conjugated secondary antibodies for 1 h at room temperature. The immunoblots were detected using ECL (Thermo) according to the manufacturer’s protocol.

### Statistical analysis

All quantitative data are presented as the mean ± SD of at least three independent experiments. Statistical significance was determined by Student’s *t* test and is expressed as a *P* value.

Normalized and log2 transformed gene expression data (RNA sequencing) of TCGA Pan-Cancer were downloaded from the Xena data hub (https://xenabrowser.net/hub/). The pathway activity score was calculated by PLAGE^61^. The correlation was tested by Spearman’s rank correlation test.

### Reporting summary

Further information on research design is available in the Nature Research Reporting Summary linked to this article.

## Supplementary information

Supplementary Information

Reporting Summary

## Data Availability

The source data underlying Figs. 1–7 as well as Supplementary Figs. 1–7 are provided as a Source Data file. All the other data supporting the findings of this study are available within the article and its supplementary information files and from the corresponding author upon reasonable request. A reporting summary for this article is available as a Supplementary Information file.

## Source data

Source Data
