# Supplementary material for: TRIM21 and PHLDA3 negatively regulate the crosstalk between the PI3K/AKT pathway and PPP metabolism
Source: Nat Commun. 2020 Apr 20;11:1880. doi: 10.1038/s41467-020-15819-3 (PMC7170963; doi:10.1038/s41467-020-15819-3)
Supplement: Supplementary file 1 — Supplementary Information [file 41467_2020_15819_MOESM1_ESM.pdf]

SUPPLEMENTARY INFORMATION

TRIM21 and PHLDA3 Negatively Regulate the Crosstalk between the PI3K/AKT Pathway and PPP Metabolism

Jie Cheng[1], Yan Huang[1]*, Xiaohui Zhang[2]*, Yue Yu[2], Shumin Wu[3], Jing Jiao[3], Linh Tran[3], Wanru Zhang[1], Ran Liu[1], Liuzhen Zhang[1], Mei Wang[1], Mengyao Wang[1], Wenyu Yan[1], Yilin Wu[1], Fangtao Chi[4], Peng Jiang[5], Xinxiang Zhang[2] and Hong Wu[1,3@]

Supplementary Figures

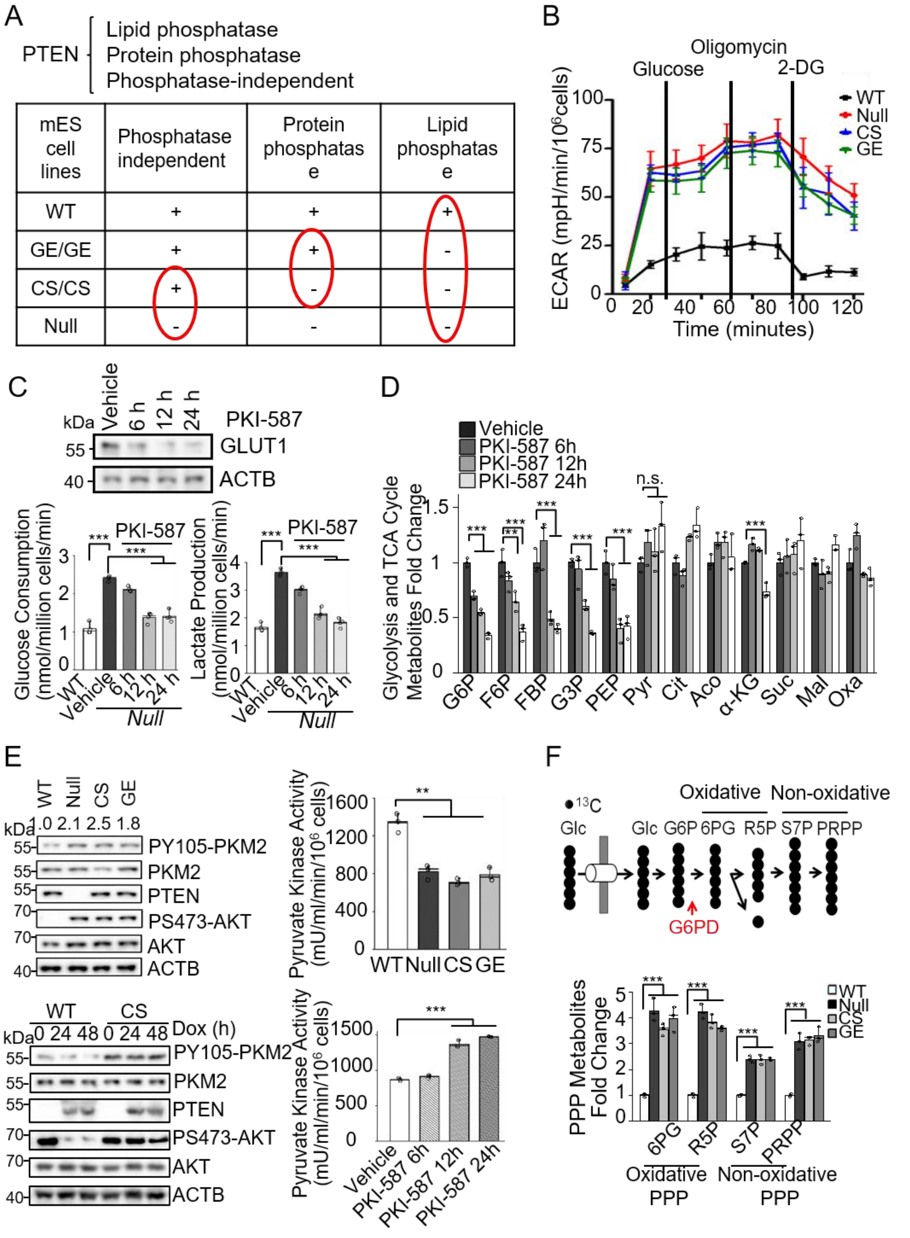

Supplementary Figure 1. PI3K activation diverts glycolytic intermediates to PPP

(A) Upper panel, major biological functions of PTEN; lower panel, genetic separation of the major biological functions of PTEN by site-specific knock-in mutations and deletions.

(B) PTEN loss of function or PI3K activation increases the ECAR rates. *Pten* WT, null, CS and GE mES cells were cultured without glucose for 1 h before the sequential addition of glucose (80 mM), oligomycin (2 mM), and 2-DG (50 mM). ECAR was calculated as the rate of PH change per minute per 2×10^4 cells.

(C) PI3K inhibition in the *Pten* null mES cells decreases the GLUT1 levels (upper

panel), glucose consumption (lower left panel) and lactate production (lower right panel) to levels comparable to those of the WT cells.

(D) PI3K inhibition decreases the levels of ^{13}C-labeled glycolytic intermediates from G6P to PEP in the *Pten* null mES cells compared to the vehicle treatment cells. (E) Loss of PTEN lipid phosphatase activity increases PY105-PKM2 (left upper panel) and decreases PKM2 activity (right upper panel), while PI3K inhibition decreases PY105-PKM2 (left lower panel) and increases PKM2 activity (right lower panel). (F) Upper panel, a schematic illustrating [U-^{13}C] glucose tracing into the PPP; lower panel, levels of labeled PPP metabolites are increased in the *Pten* null, CS, and GE mES cells compared to the WT cells.

Cell extracts were prepared and analyzed using LC-MS. Data are presented as fold change and the mean ± SD and were compared to WT cells. Each experiment was performed n=4 (C) and n=3 (D, E, F) independent times. * $p < 0.05$, ** $p < 0.001$, and *** $p<0.001$, based on Student's *t*-test (two-sided ANOVA).

Source data are provided as a Source Data file.

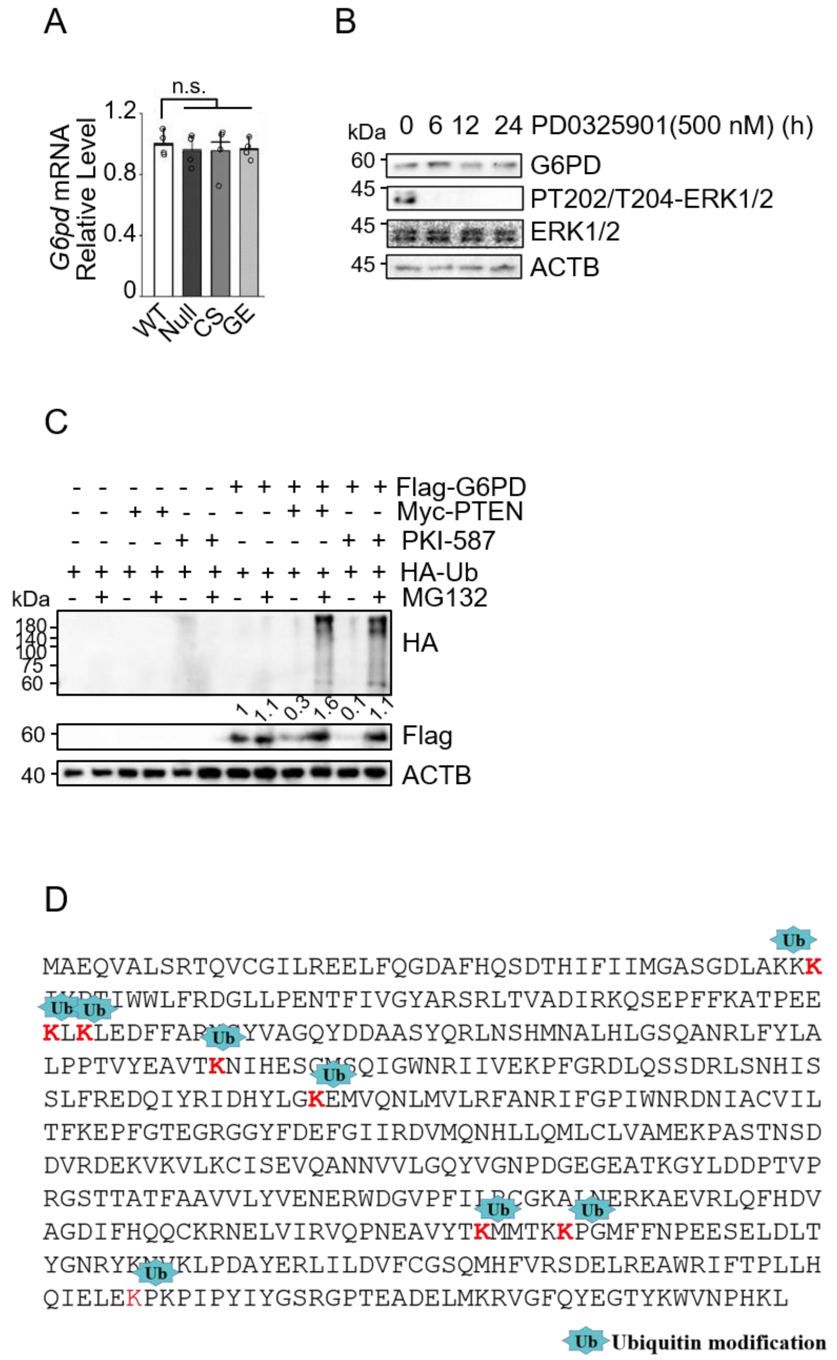

Supplementary Figure 2. PI3K/AKT activation promotes PPP by stabilizing G6PD

(A) *G6pd* mRNA levels are not changed in the *Pten* WT, null, CS and GE mES cells.
(B) The G6PD protein levels remained unchanged in the PC3 cells treated with PD0325901 (500 nM) for the indicated time periods.

(C) HEK293T cells were co-transfected with *HA-ubiquitin*, *3×Flag-G6PD* and *Myc-PTEN* expression plasmids. Twenty-four hours later, the cells were incubated with or without MG132 (5 μM) or PKI-587 (1 μM) for 16 h. Total cell lysates were

harvested, immunoprecipitated with a Flag antibody and immunoblotted with a HA antibody and followed by Western blot analysis using the indicated antibodies.

(D) The potential G6PD ubiquitination-modified sites identified by affinity MS.

Data are presented as fold changes and the mean ± SD and were compared to WT cells. Each experiment was performed n=4 independent times. * $p < 0.05$, ** $p < 0.001$, and *** $p<0.001$, based on Student's *t*-test (two-sided ANOVA).

Source data are provided as a Source Data file.

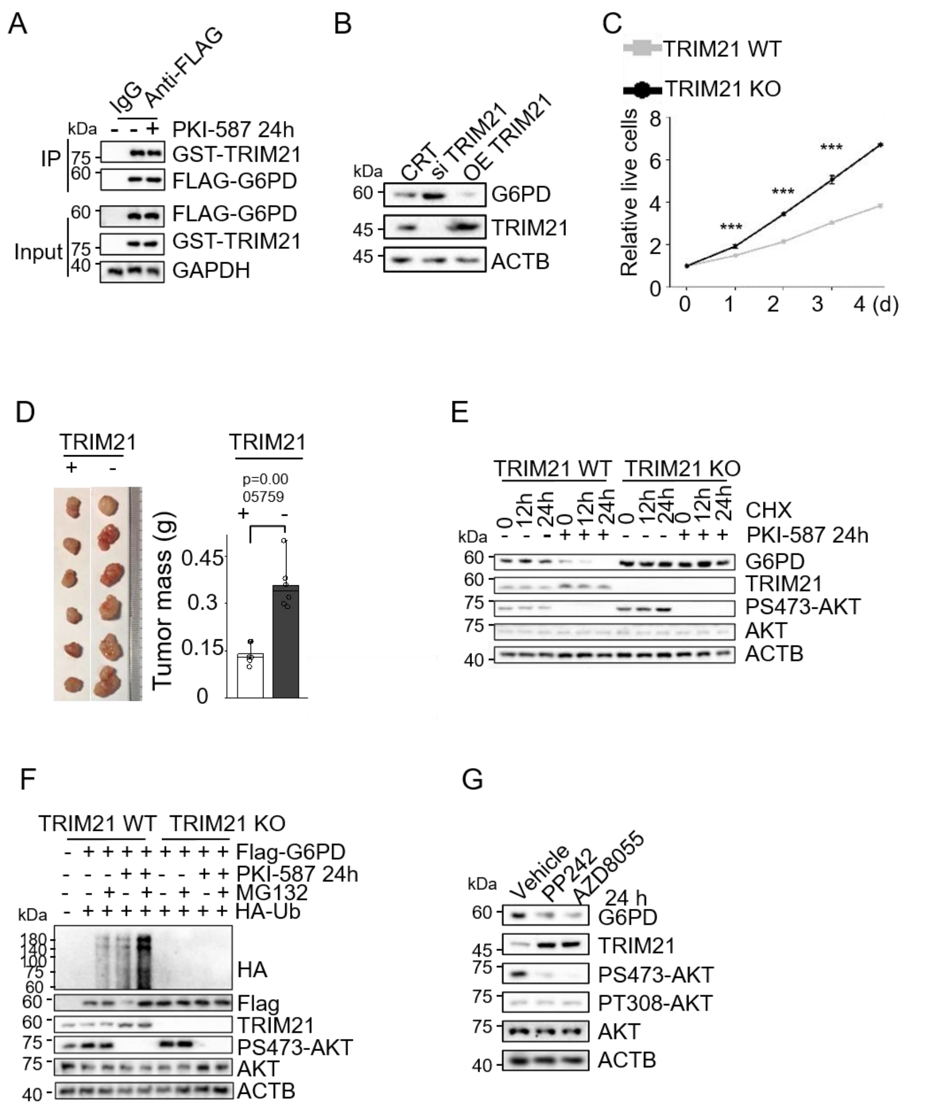

Supplementary Figure 3. TRIM21 is responsible for PI3K/AKT-regulated G6PD stability

(A) PI3K inhibition does not influence the interaction between exogenously expressed TRIM21 and G6PD proteins. HEK293T cells cotransfected with *Flag-G6PD* and *GST-TRIM21* expression plasmids were incubated with or without PKI-587 (1 μM) for 24 h. Total cell lysates were immunoprecipitated with a Flag antibody and immunoblotted with the indicated antibodies.

(B) TRIM21 controls the G6PD protein levels. HEK293T cells were transfected with scramble siRNA, siTRIM21, or *FLAG-TRIM21* expression plasmids and cultured for two days. Cell lysates were subjected to Western blot analysis using the indicated antibodies.

(C) *TRIM21* knockout leads to increased cell growth *in vitro*. Equal numbers of

isogenic *TRIM21* WT and knockout A549 cells were seeded in 96-well plates at 2,000 cells/well. Relative cell viabilities were tested using a CCK8 kit and are presented as growth curves.

(D) *TRIM21* knockout leads to increased cell growth *in vivo*. Equal numbers of isogenic *TRIM21* WT and knockout A549 cells were implanted into the bilateral flanks of nude mice. Tumor mass was measured and calculated.

(E) TRIM21 controls G6PD stability. The half-lives of G6PD in the isogenic *TRIM21* WT and knockout A549 cells were measured after vehicle or PKI-587 (1 μM) treatment for 24 h with CHX (100 μg/ml) treatment for the indicated times. Cell lysates were subjected to Western blot analysis using the indicated antibodies.

(F) TRIM21 controls G6PD ubiquitination. The isogenic *TRIM21* WT and knockout A549 cells were cotransfected with indicated plasmids. 24 hours later, cells were incubated with or without MG132 (5 μM) for 16 h or PKI-587 (1 μM) for 24 h. Total cell lysates were immunoprecipitated with a Flag antibody and immunoblotted with an HA antibody, followed by Western blot analysis using the indicated antibodies.

(G) Dual mTOR inhibitors regulate the TRIM21 and G6PD protein levels. PC3 cells were treated with PP242 (5 μM) or AZD8055 (2 μM) for 24 h. Cell lysates were subjected to immunoblotting with the indicated antibodies.

Data are presented as fold changes and the mean ± SD and were compared to control cells. A-C and E-G: n=3, each experiment was performed at least three independent times.; D: n=6 independent xenografts. * $p < 0.05$, ** $p < 0.001$, and *** $p<0.001$, based on Student's *t*-test (two-sided ANOVA).

Source data are provided as a Source Data file.

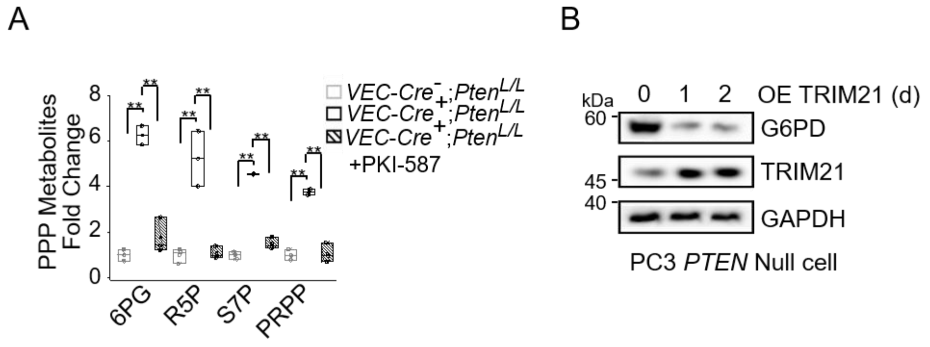

C

Cancer type	Rho	p value
Thyroid carcinoma	-0.63	<0.01
Testicular germ cell tumor	-0.61	<0.01
Diffuse large B-cell lymphoma	-0.55	<0.01
Skin cutaneous melanoma	-0.54	<0.01

Supplementary Figure 4. PI3K/AKT regulates TRIM21 and PPP *in vivo* and in human cancers

(A) PI3K regulates the PPP metabolic pathways in the *Pten* null T-ALL mouse model by box plot. (B) TRIM21 overexpression decreases the G6PD protein levels. The *PTEN* null PC3 cells were transfected with *FLAG-TRIM21* expression plasmids. Cell lysates were subjected to Western blot analysis using the indicated antibodies at the indicated times.

(C) Correlations between the PI3K pathway activity score and the TRIM21 mRNA expression levels in human cancers (based on TCGA data).

Cell extracts were prepared and analyzed using LC-MS. Data are presented as fold changes and the mean ± SD and were compared to WT cells. A: n=3 independent mouse samples; B: n=3 independent experiments. * $p < 0.05$, ** $p < 0.001$, and *** $p<0.001$, based on Student's *t*-test (two-sided ANOVA).

Source data are provided as a Source Data file.

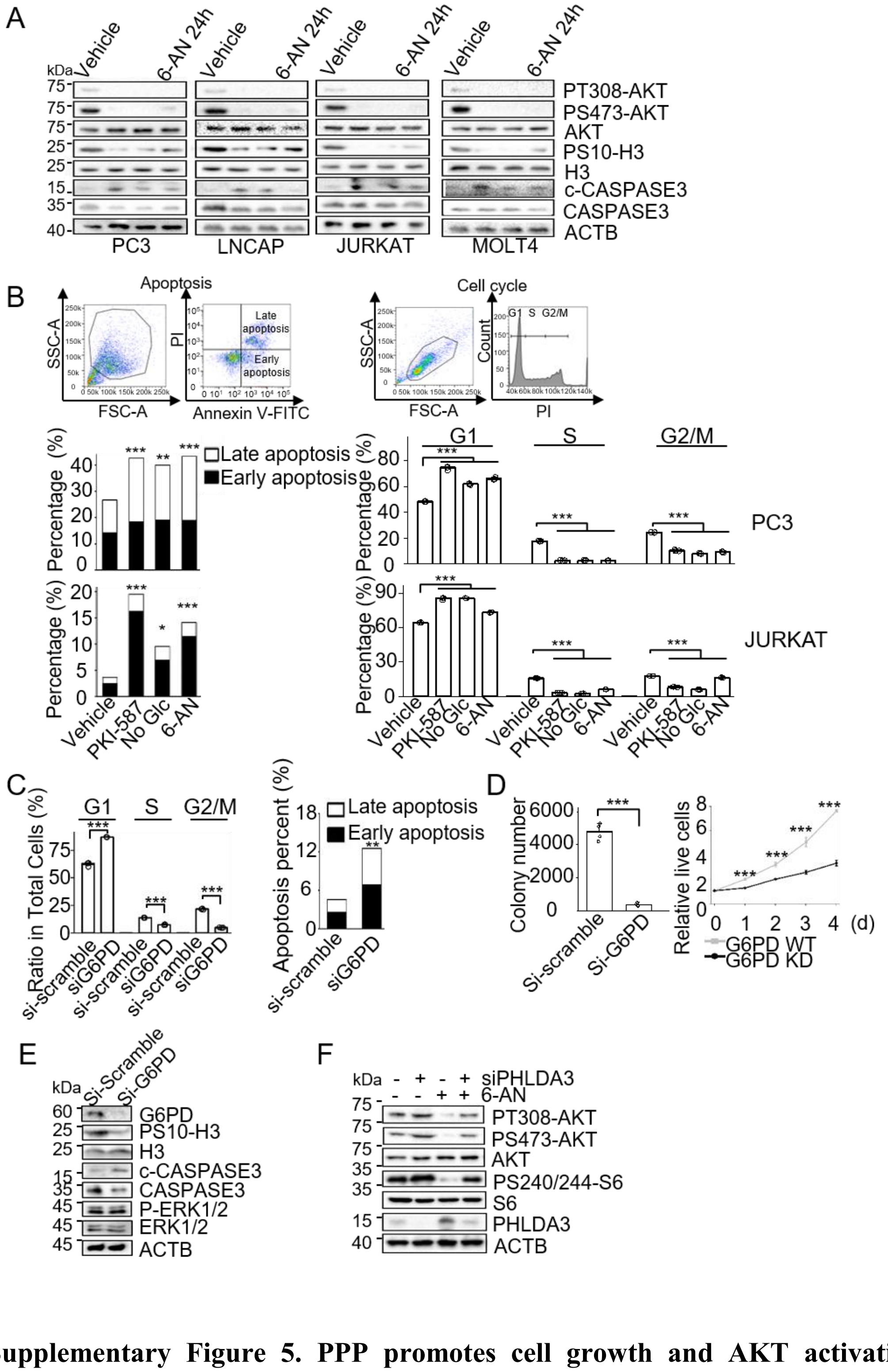

Supplementary Figure 5. PPP promotes cell growth and AKT activation by inhibiting PHLDA3

(A-B) The PPP activates AKT and supports cell growth in human cancer cell lines. PC3, LNCAP, JURKAT and MOLT4 cells were treated with PKI-587 (1 μM) or 6-AN (100 nM) for 24 h or cultured in glucose-depleted medium for 12 h. Cell lysates were subjected to immunoblotting with the indicated antibodies (A). For PPP-regulated cell

proliferation and survival, PC3 and JURKAT cells were treated with PKI-587 (1 μM) or 6-AN (100 nM) for 24 h or cultured in glucose-depleted medium for 24 h. The percentages of apoptotic cells and cells in each phase of the cell cycle were determined by FACS analysis (B).

(C-E) G6PD knockdown in PC3 cells inhibits cell growth. *G6PD* was knocked down by siG6PD RNA. Forty-eight hours later, the growth and survival of si-scramble control and siG6PD knockdown cells were measured to determine the percentages of cells in each phase of the cell cycle and assess apoptosis via FACS analysis (C); colony number and relative live cells were measured by trypan blue or CCK8 assays (D); cell lysates were subjected to immunoblotting with the indicated antibodies (E).

(F) *PHLDA3* knockdown blocks 6-AN (100 nM) treatment-induced AKT inactivation in PC3 cells.

Data are presented as fold changes and the mean ± SD and were compared to untreated cells. Each experiment was performed n=4 (B, C) and n=3 (D) independent times. * $p < 0.05$, ** $p < 0.001$, and *** $p<0.001$, based on Student's *t*-test (two-sided ANOVA).

See also Supplementary Table 2. Source data are provided as a Source Data file.

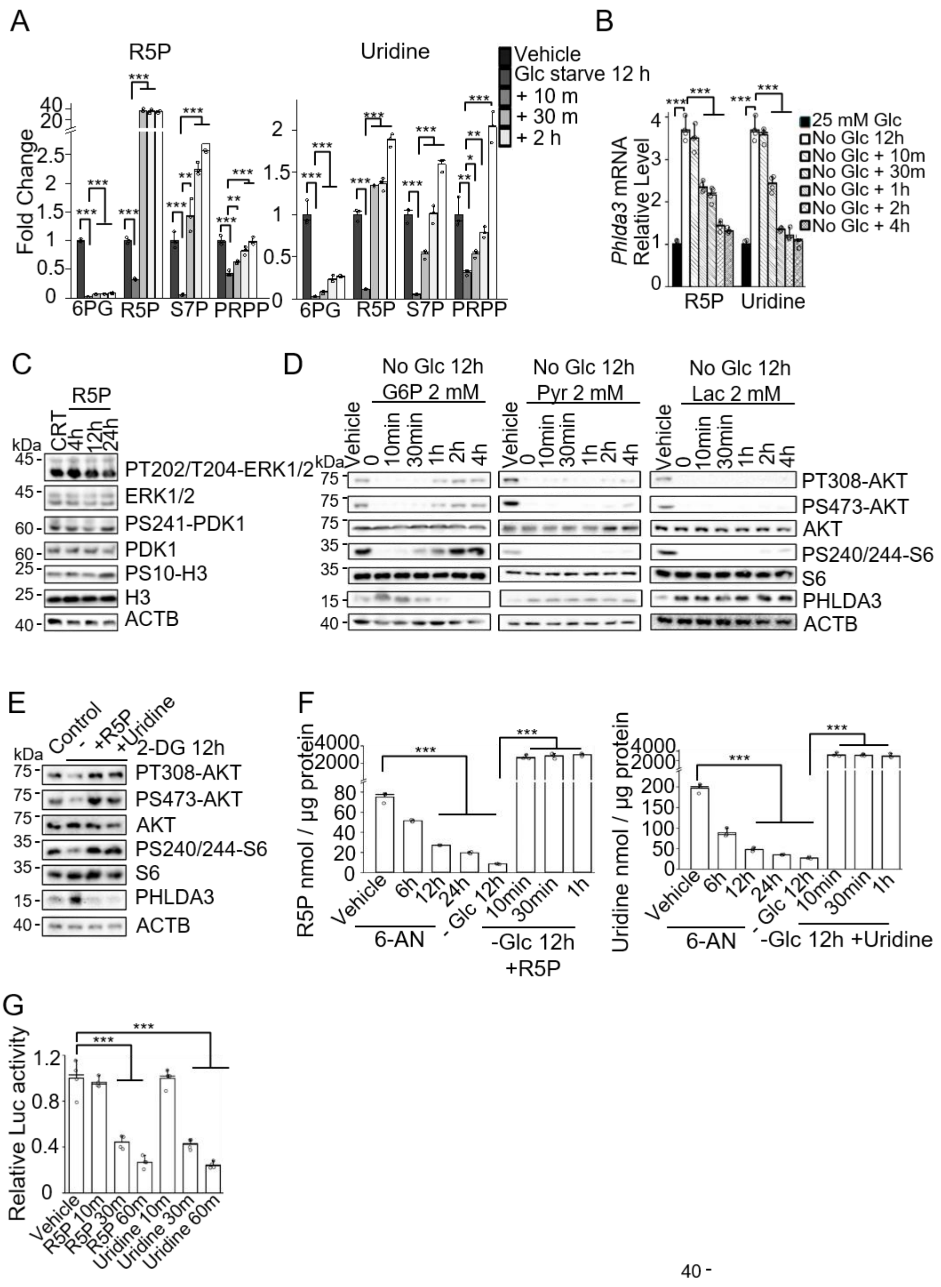

Supplementary Figure 6. PPP metabolites promote AKT activation by inhibiting PHLDA3

(A-B) Exogenous R5P or uridine could immediately incorporate into PPP metabolites. PC3 cells were treated with 2 mM R5P or uridine for the indicated time periods after 12 h of glucose starvation. The PPP metabolites and *PHLDA3* mRNA levels were measured.

(C) R5P treatment had no effect on the ERK and PDK1 pathways. The *Pten* null mES cells were treated with R5P for the indicated time periods. Cell lysates were subjected to immunoblotting with the indicated antibodies.

(D) The effects of G6P (left panel), pyruvate (middle panel) and lactate (right panel) on AKT activation and PHLDA3 protein levels. The *Pten* null mES cells were treated with 2 mM G6P, 10 mM pyruvate, or 25 mM lactate for the indicated times after 12 h of glucose starvation. Cell lysates were subjected to immunoblotting with the indicated antibodies.

(E) 2-DG treatment cannot decrease the R5P and uridine-induced AKT activation. The *Pten* null mES cells were treated with R5P or uridine for 2 h after 12 h of 2-DG (10 mM) treatment. Cell lysates were subjected to immunoblotting with the indicated antibodies.

(F) The *Pten* null mES cells were treated with 6-AN for the indicated time periods or glucose starvation for 12 h, and then, R5P or uridine was added to the media for the indicated time periods before measurements.

(G) The *PHLDA3* promoter-luciferase construct responded to R5P and uridine supplementation in a time-dependent manner. PC3 cells were transfected with the reporter construct and 24 h later supplemented with R5P or uridine for 1 h. The cell lysates were harvested for the luciferase assays.

Cell extracts were prepared and analyzed using LC-MS. Data are presented as fold changes and the mean ± SD and were compared to untreated cells. Each experiment was performed n=4 (B, G) and n=3 (A, F) independent times. $* p < 0.05$, $** p < 0.001$, and $*** p<0.001$, based on Student's *t*-test (two-sided ANOVA).

Source data are provided as a Source Data file.

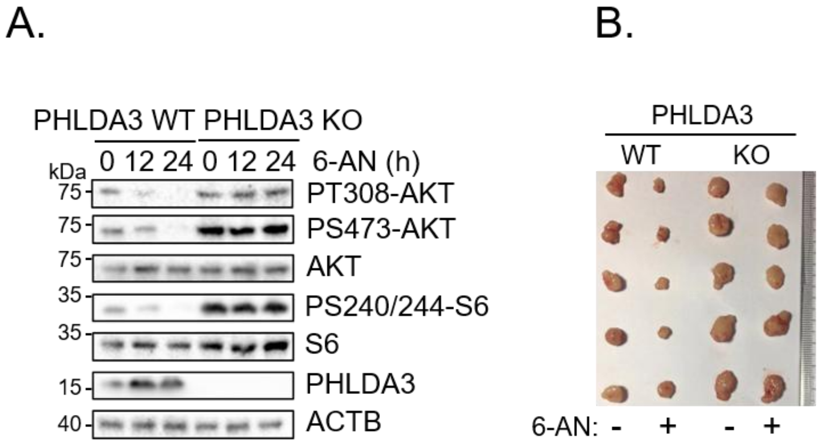

Supplementary Figure 7. The PPP controls cell proliferation and survival *in vivo*

(A) The effects of 6-AN treatment-induced PHLDA3 upregulation and AKT inhibition are blocked by PHLDA3 knockout. n=3 independent experiments.

(B) *PHLDA3* knockout blocks 6-AN-induced cell growth *in vivo*. Equal numbers of *PHLDA3* WT and *PHLDA3* knockout cells were implanted onto the bilateral flanks of nude mice. Tumors were isolated at the end points and photographed. n=5 independent xenografts.

Source data are provided as a Source Data file.

Supplementary Tables: Lists of inhibitors, cancer cell lines and essential experimental resources used in this study.

Supplementary Table 1: A list of the inhibitors used in this study.

Drug	Target	IC50
PKI-587	PI3K/mTORC1	PI3Kα =0.4 nM; PI3Kβ, δ, γ＞0.4 nM; mTOR =1.0 nM.
BAY1082439	PI3K	PI3Kα =5 nM; PI3Kβ =15 nM; PI3Kδ =1 Nm; PI3Kγ =52 nM.
GDC-0068	Pan-AKT	Akt1 =5 nM; Akt2 =18 nM; Akt3 =8 nM
GDC-0980	PI3K/mTORC1	PI3Kα =5 nM; PI3Kβ =27 nM; PI3Kδ =7 Nm; PI3Kγ =14 nM; mTOR =17 nM.
Rapamycin	mTORC1	mTOR ＜0.1 nM
PD0325901	MEK	0.33 nM
6-Aminonicotinamide	G6PD, PGD	Ki =0.46 μM
PP242	mTORC1 and mTORC2	Ki =8 nM
AZD8055	mTORC1 and mTORC2	Ki =0.8 nM

Supplementary Table 2: A list of the human cancer cell lines used in this study.

Cancer type	Prostate cancer		T-ALL					
Cell line	LNCAP	PC3	CEM	MOLT3	MOLT4	MOLT16	KE-37	JURKAT
PTEN	Mut	Null	Null	Null	Null	Null	Null	Null
p53	WT	Null	Null	WT	Null	Mut	Null	Null
MYC	WT	WT	WT	WT	WT	TCRα-c-myc	TCRα-c-myc	WT

Supplementary Table 3: A list of essential materials used in this study.

Reagent	Designation	Identifiers	Additional information
Antibody	GLUT1	Cell Signaling Technology 12939	1:1000 for WB
Antibody	PTEN	Cell Signaling Technology 9188	1:1000 for WB
Antibody	PKM2	Cell Signaling Technology 4053	1:1000 for WB
Antibody	P-PKM2(Tyr105)	Cell Signaling Technology 3827	1:1000 for WB
Antibody	AKT	Cell Signaling Technology 9272	1:1000 for WB
Antibody	Phospho-AKT (Ser473)	Cell Signaling Technology 4060	1:1000 for WB
Antibody	Phospho-AKT (Thr308)	Cell Signaling Technology 13038	1:1000 for WB
Antibody	S6	Cell Signaling Technology 2217	1:1000 for WB
Antibody	Phospho-S6 (Ser240/244)	Cell Signaling Technology 5364	1:1000 for WB
Antibody	GST	Cell Signaling Technology 2624	1:1000 for WB
Antibody	Histone H3	Cell Signaling Technology 4499	1:1000 for WB
Antibody	Phospho-Histone H3 (Ser10)	Cell Signaling Technology 53348	1:1000 for WB
Antibody	CASPASE 3	Cell Signaling Technology 9665	1:1000 for WB
Antibody	Cleaved CASPASE 3	Cell Signaling Technology 9664	1:1000 for WB
Antibody	TRIM21	Santa Cruz sc-25351	1:1000 for WB
Antibody	ACTB	Santa Cruz sc-1616	1:1000 for WB
Antibody	G6PD	Sigma HPA000834	1:1000 for WB
Antibody	Ki67	Abcam ab15580	1:1000 for WB
Antibody	FLAG M2	Sigma F3165	1:1000 for WB
Antibody	HA	Sigma H6908	1:1000 for WB
Antibody	PHLDA3	Abcam ab81464	1:1000 for WB
Antibody	G6PD	Abcam ab993	1:1000 for WB
Antibody	PT202/T204-ERK1/2	Cell Signaling Technology 4370S	1:1000 for WB
Antibody	ERK	Cell Signaling Technology 9102	1:1000 for WB
Antibody	PS241-PDK1	Cell Signaling Technology 3438	1:1000 for WB
Antibody	PDK1	Cell Signaling Technology 13037S	1:1000 for WB
Antibody	IRF3	Cell Signaling Technology 11904	1:1000 for WB
Antibody	HRP-conjugated secondary antibodies	Jackson ImmunoResearch Laboratories	1:1000 for WB
Antibody	Anti-Mouse IgG, light chain specific HRP-conjugated secondary antibodies	Jackson ImmunoResearch Laboratories115-035-174	1:5000 for WB
Antibody	Mouse IgG	Santa Cruz Sc-2025	1:400 for co-IP
Antibody	Rabbit IgG	Santa Cruz Sc-2027	1:400 for co-IP

Beads	nProtein A Sepharose™ 4 Fast Flow	GE Healthcare 17-5280-01	1:100 for co-IP
Chemical compound, drug	Trichloroacetic acid	Sigma	T9159
Chemical compound, drug	6-Aminonicotinamide (6-AN)	Sigma	A68203
Chemical compound, drug	CHX	Sigma	C7698
Chemical compound, drug	MG132	TargetMol	T2154
Chemical compound, drug	Doxorubicin (Dox)	Sigma	D1515
Chemical compound, drug	PKI-587	Pfizer	PF-05212384
Chemical compound, drug	BAY1082439	Bayer Health Sciences	1375469-38-7
Chemical compound, drug	GDC-0980	Selleckchem	S2696
Chemical compound, drug	GDC-0068	Selleckchem	S2808
Chemical compound, drug	Rapamycin	Selleckchem	S1039
Chemical compound, drug	PFTα	Sigma	P4359
Chemical compound, drug	PD0325901	StemCell Technologies	72184
Chemical compound, drug	2-Deoxy-D-glucose	Sigma	D8375-10MG
Chemical compound, drug	PP242	Selleck	S2218
Chemical compound, drug	AZD8055	Selleck	S1555
Chemical compound, drug	Streptolysin O	Sigma	S5265
Chemical compound, drug	U-13C6 glucose	Cambridge Isotype Lab	CLM-1396-0.5
Chemical compound, drug	1,2-13C2 glucose	Cambridge Isotype Lab	CLM-504-0.5
