# Supplementary material for: TRIM21 and PHLDA3 negatively regulate the crosstalk between the PI3K/AKT pathway and PPP metabolism
Source: Nat Commun. 2020 Apr 20;11:1880. doi: 10.1038/s41467-020-15819-3 (PMC7170963; doi:10.1038/s41467-020-15819-3)
Supplement: Supplementary file 2 — Reporting Summary [file 41467_2020_15819_MOESM2_ESM.pdf]

nature research

Corresponding author(s): Hong Wu

Last updated by author(s): Mar 15, 2020

# Reporting Summary

Nature Research wishes to improve the reproducibility of the work that we publish. This form provides structure for consistency and transparency in reporting. For further information on Nature Research policies, see Authors & Referees and the Editorial Policy Checklist .

## Statistics

For all statistical analyses, confirm that the following items are present in the figure legend, table legend, main text, or Methods section.

| n/a | Confirmed | |
|---|---|---|
| ☐ | ☒ | The exact sample size (*n*) for each experimental group/condition, given as a discrete number and unit of measurement |
| ☐ | ☒ | A statement on whether measurements were taken from distinct samples or whether the same sample was measured repeatedly |
| ☐ | ☒ | The statistical test(s) used AND whether they are one- or two-sided<br>*Only common tests should be described solely by name; describe more complex techniques in the Methods section.* |
| ☒ | ☐ | A description of all covariates tested |
| ☐ | ☒ | A description of any assumptions or corrections, such as tests of normality and adjustment for multiple comparisons |
| ☐ | ☒ | A full description of the statistical parameters including central tendency (e.g. means) or other basic estimates (e.g. regression coefficient) AND variation (e.g. standard deviation) or associated estimates of uncertainty (e.g. confidence intervals) |
| ☐ | ☒ | For null hypothesis testing, the test statistic (e.g. *F*, *t*, *r*) with confidence intervals, effect sizes, degrees of freedom and *P* value noted<br>*Give P values as exact values whenever suitable.* |
| ☒ | ☐ | For Bayesian analysis, information on the choice of priors and Markov chain Monte Carlo settings |
| ☒ | ☐ | For hierarchical and complex designs, identification of the appropriate level for tests and full reporting of outcomes |
| ☐ | ☒ | Estimates of effect sizes (e.g. Cohen's *d*, Pearson's *r*), indicating how they were calculated |

*Our web collection on statistics for biologists contains articles on many of the points above.*

## Software and code

Policy information about availability of computer code

| | |
|---|---|
| Data collection | Softmax pro 6.3 was used to collect fluorescent and absorption data for enzyme assay.<br>Perkinelmer 2030 manager was used for luciferase signal collection.<br>Seahorse XFe24 was used for ECR detection.<br>Real-Time PCR Analysis Software CFX Maestro Software was used for Real-Time PCR signal collection.<br>Image lab 5.0 was used for western blot and colony signal collection.<br>Xcalibur Software 2.2.0 was used for metabolism signal collection.<br>BD FACSuite Flow Cytometry Software was used for FACS signal collection. |
| Data analysis | OriginPro 2018C b9.5.0.193 was used for data plotting and statistical analysis.<br>Image lab 5.0 was used for western blot data and colony formation analyzing.<br>Xcalibur Software 2.2.0 was used for metabolism data acquisition and processing.<br>Flowjo V10 was used for FACS data analyzing.<br>Statistical analyses were conducted in R v3.6.0.<br>Pathway activity score was calculated by R package GSVA V1.20.0. |

For manuscripts utilizing custom algorithms or software that are central to the research but not yet described in published literature, software must be made available to editors/reviewers. We strongly encourage code deposition in a community repository (e.g. GitHub). See the Nature Research guidelines for submitting code & software for further information.

## Data

Policy information about availability of data

All manuscripts must include a data availability statement. This statement should provide the following information, where applicable:

- Accession codes, unique identifiers, or web links for publicly available datasets
- A list of figures that have associated raw data
- A description of any restrictions on data availability

The source data underlying Figure 1 - 7 as well as Supplementary Figure 1 - 7 are provided as a Source Data file. All the other data supporting the findings of this study are available within the article and its supplementary information files and from the corresponding author upon reasonable request.
The TCGA database used in the study was along with appropriately accessible links.

# Field-specific reporting

Please select the one below that is the best fit for your research. If you are not sure, read the appropriate sections before making your selection.

☒ Life sciences ☐ Behavioural & social sciences ☐ Ecological, evolutionary & environmental sciences

For a reference copy of the document with all sections, see nature.com/documents/nr-reporting-summary-flat.pdf

# Life sciences study design

All studies must disclose on these points even when the disclosure is negative.

| | |
|---|---|
| Sample size | Statistical methods were not used to predetermine sample size. All sample size was at least three independent replicates. Animal and patient sample size was determined by experimental feasibility and sample availability to demonstrate certain results. |
| Data exclusions | No data were excluded from the analyses. |
| Replication | Biological replications (three biological replicates at least) and statistics were indicated in the legends. All attempts at replication were successful based on replications on different days showing comparable significance level for biological comparison. |
| Randomization | Samples were allocated into experimental groups by the confirmed genetic modification of the cell line (e.g. CRISPR-Cas9 deletion, siRNA knocking down, doxycycline-induced protein expression) and/or culturing conditions (e.g. isotope tracing). Mouse samples were allocated into experimental groups by the confirmed Pten genetic deletion. This design does not allow for randomization, as the origin of samples is critical. However, whenever possible samples were analyzed in a randomized order (i.e. when run on liquid-chromatography mass spectrometry). |
| Blinding | The researchers were blinded during animals research data collection, experiments apart from animal studies, and data analysis. |

# Reporting for specific materials, systems and methods

We require information from authors about some types of materials, experimental systems and methods used in many studies. Here, indicate whether each material, system or method listed is relevant to your study. If you are not sure if a list item applies to your research, read the appropriate section before selecting a response.

### Materials & experimental systems

| n/a | Involved in the study | |
|---|---|---|
| ☐ | ☒ | Antibodies |
| ☐ | ☒ | Eukaryotic cell lines |
| ☒ | ☐ | Palaeontology |
| ☐ | ☒ | Animals and other organisms |
| ☒ | ☐ | Human research participants |
| ☒ | ☐ | Clinical data |

### Methods

| n/a | Involved in the study | |
|---|---|---|
| ☒ | ☐ | ChIP-seq |
| ☐ | ☒ | Flow cytometry |
| ☒ | ☐ | MRI-based neuroimaging |

## Antibodies

| | |
|---|---|
| Antibodies used | Antibody GLUT1 Cell Signaling Technology (12939),1:1000 for WB<br>Antibody PTEN Cell Signaling Technology (9188),1:1000 for WB<br>Antibody PKM2 Cell Signaling Technology (4053),1:1000 for WB<br>Antibody Phospho-PKM2(Tyr105) Cell Signaling Technology (3827),1:1000 for WB<br>Antibody AKT Cell Signaling Technology (9272),1:1000 for WB<br>Antibody Phospho-AKT (Ser473) Cell Signaling Technology (4060),1:1000 for WB,1: 800 for IHC<br>Antibody Phospho-AKT (Thr308) Cell Signaling Technology (13038),1:1000 for WB |

| | |
|---|---|
| | Antibody S6 Cell Signaling Technology (2217),1:1000 for WB<br>Antibody GST Cell Signaling Technology (2624),1:1000 for WB<br>Antibody Histone H3 Cell Signaling Technology (4499),1:1000 for WB<br>Antibody Phospho-Histone H3 (Ser10) Cell Signaling Technology (53348),1:1000 for WB<br>Antibody CASPASE 3 Cell Signaling Technology (9665),1:1000 for WB<br>Antibody Cleaved CASPASE 3 Cell Signaling Technology (9664),1:1000 for WB<br>Antibody TRIM21 Santa cruz (sc-25351),1:100 for co-IP, 1:1000 for WB<br>Antibody ACTB Santa cruz (sc-1616,)1:2000 for WB<br>Antibody FLAG M2, Sigma (F3165),1:5000 for WB<br>Antibody HA ,Sigma (H6908,)1:5000 for WB<br>Antibody PHLDA3, Abcam (ab81464),1:1000 for WB<br>Antibody G6PD, Abcam (ab993),1:400 for co-IP<br>Antibody PT202/T204-ERK1/2, Cell Signaling Technology (4370S),1:1000 for WB<br>Antibody ERK, Cell Signaling Technology (9102),1:1000 for WB<br>Antibody PS241-PDK1, Cell Signaling Technology (3438),1:1000 for WB<br>Antibody PDK1, Cell Signaling Technology (13037S),1:1000 for WB<br>Antibody IRF3, Cell Signaling Technology (11904),1:1000 for WB<br>Antibody HRP-conjugated secondary antibodies Jackson ImmunoResearch Laboratories (111-035-003)1:5000 for WB<br>Antibody Anti-Mouse IgG, santa cruz (Sc-2025), 1:400 for co-IP<br>Antibody Anti-Rabbit IgG, santa cruz (Sc-2027), 1:400 for co-IP<br>Antibody Anti-Mouse IgG, light chain specific HRP-conjugated secondary antibodies Jackson ImmunoResearch Laboratories (115-035-174), 1:5000 for WB |
| Validation | All western blot an IP antibodies in the manuscript had been validated. Each primary antibody data provided in the manuscript has been validated for the species and application on the manufacturer's website. |

## Eukaryotic cell lines

Policy information about cell lines

| | |
|---|---|
| Cell line source(s) | T-ALL cells (JURKAT, CEM, KE-37, MOLT3, MOLT4 and MOLT16) , human prostate cancer cells (PC3 and LNCAP) and HEK293T cells were ordered from ATCC. Isogenic TRIM21-WT and -knockout A549 cells, PHLDA3-WT and -knockout HeLa cells were generously provided by Dr. Wensheng Wei of Peking University. Pten WT, null and mutant (CS and GE) mES cells and PC3 WT/CS-PTEN-inducible cells were established by our lab. |
| Authentication | Cell lines used in this study have not been authenticated after purchasing from vendors.<br>Pten WT, null and mutant (CS and GE) mES cells, PC3 WT/CS-PTEN-inducible cells, and CRISPR knockout cells were authenticated by immunoblotting of targeted proteins and Sanger sequencing of modification region of targeted genes. |
| Mycoplasma contamination | All cells used have been tested negative for mycoplasma contamination. |
| Commonly misidentified lines<br>(See ICLAC register) | No such cell lines were used. |

## Animals and other organisms

Policy information about studies involving animals; ARRIVE guidelines recommended for reporting animal research

| | |
|---|---|
| Laboratory animals | The generation of the Pb-Cre+;PtenloxP/loxP prostate cancer model (male, 10 weeks) and VEC-Cre+;PtenloxP/loxP T-ALL model (male or female, 8 weeks) has been described previously (Guo, W. et al. Nature, 2008)(Wang, S. et al. Cancer cell, 2003).<br>Male CAnN.Cg-Foxn1nu/CrlVr mice (6 weeks) were purchased from Charles River Laboratories China. |
| Wild animals | The study did not involve wild animals. |
| Field-collected samples | The study did not involve field-collected samples |
| Ethics oversight | Animal housing, breeding, and surgical procedures were approved by the Ethics Committee under ID LSC-WuH-1 and conducted in accordance with the regulations of the Division of Laboratory Animal Medicine at Peking University. |

Note that full information on the approval of the study protocol must also be provided in the manuscript.

## Flow Cytometry

### Plots

Confirm that:

☒ The axis labels state the marker and fluorochrome used (e.g. CD4-FITC).

☒ The axis scales are clearly visible. Include numbers along axes only for bottom left plot of group (a 'group' is an analysis of identical markers).

☒ All plots are contour plots with outliers or pseudocolor plots.

☒ A numerical value for number of cells or percentage (with statistics) is provided.

### Methodology

| | |
|---|---|
| Sample preparation | Cells were dissected with 0.25% trypsin. Single cell suspension was made in 20mM HEPES (Sigma-Aldrich) and filtered through 40 µm cell strainer, single cells were resuspended with PBS containing 1% FBS and 0.5% BSA. |
| Instrument | Cells were sorted on BD Accuri C6 flow cytometer (BD Biosciences, San Jose, CA, USA) . |
| Software | BD FACSuite Flow Cytometry Software was used for signal collection. Flowjo V10 was used for data analysis. |
| Cell population abundance | Cell purity was 99.3% determined by post-sort purity checks of representative samples. |
| Gating strategy | Cells were first gated using FSC/SSC characteristics, and doublets were sequentially excluded by comparing FSC- and SSC-height and -area signals. Specifically,  apoptosis cells were identified as Annexin V and PI and the cell cycle of cells were identified as PI. |

☒ Tick this box to confirm that a figure exemplifying the gating strategy is provided in the Supplementary Information.
